# Blueberry Juice Attenuates Pulmonary Fibrosis *via* Blocking the TGF-β1/Smad Signaling Pathway

**DOI:** 10.3389/fphar.2022.825915

**Published:** 2022-03-28

**Authors:** Yali Li, Liqun Wang, Qianyu Zhang, Li Tian, Cailing Gan, Hongyao Liu, Wenya Yin, Tinghong Ye

**Affiliations:** ^1^ Sichuan University-University of Oxford Huaxi Joint Centre for Gastrointestinal Cancer, Frontiers Science Center for Disease-Related Molecular Network, State Key Laboratory of Biotherapy, West China Hospital, Sichuan University, Chengdu, China; ^2^ West China School of Public Health and West China Fourth Hospital, Sichuan University, Chengdu, China; ^3^ Prenatal Diagnosis Center, The Third Affiliated Hospital of Zhengzhou University—Maternal and Child Health Hospital of Henan Province, Zhengzhou, China

**Keywords:** blueberry juice, idiopathic pulmonary fibrosis, TGF-β1/Smad signaling pathway, epithelial–mesenchymal transition, reactive oxygen species

## Abstract

Idiopathic pulmonary fibrosis (IPF) is a progressive, fatal, and chronic lung disease, lacking a validated and effective therapy. Blueberry has demonstrated multiple pharmacological activities including anti-inflammatory, antioxidant, and anticancer. Therefore, the objective of this study was to investigate whether blueberry juice (BBJ) could ameliorate IPF. Experiments *in vitro* revealed that BBJ could significantly reduce the expressions of TGF-β1 modulated fibrotic protein, which were involved in the cascade of fibrosis in NIH/3T3 cells and human pulmonary fibroblasts. In addition, for rat primary lung fibroblasts (RPLFs), BBJ promoted the cell apoptosis along with reducing the expressions of α-SMA, vimentin, and collagen I, while increasing the E-cadherin level. Furthermore, BBJ could reverse epithelial–mesenchymal transition (EMT) phenotypic changes and inhibit cell migration, along with inducing the upregulation of E-cadherin in A549 cells. Compared with the vehicle group, BBJ treatment alleviated fibrotic pathological changes and collagen deposition in both bleomycin-induced prevention and treatment pulmonary fibrosis models. In fibrotic lung tissues, BBJ remarkably suppressed the expressions of collagen I, α-SMA, and vimentin and improved E-cadherin, which may be related to its inhibition of the TGF-β1/Smad pathway and anti-inflammation efficacy. Taken together, these findings comprehensively proved that BBJ could effectively prevent and attenuate idiopathic pulmonary fibrosis *via* suppressing EMT and the TGF-β1/Smad signaling pathway.

## 1 Introduction

Idiopathic pulmonary fibrosis (IPF) is recognized widely as a fatal, chronic, and irreversible devastating interstitial lung disease. And it is characterized by the destruction of the lung parenchyma and fibrotic remodeling of the lung tissue (Wynn, 2007). There are over 150,000 patients in the United States, approximately 5,000 new cases in the United Kingdom, and more than five million people in the world who are suffering from IPF annually (Beers and Morrisey, 2011; Navaratnam et al., 2011; Thannickal et al., 2014). Several studies have uncovered the risk factors, such as aging, cigarette smoking, environmental exposures, and virus infections (Baumgartner et al., 1997; Hubbard, 2001; Taskar and Coultas, 2006; Raghu et al., 2011). The accurate prevalence of IPF in China is still unclear. However, considering these ubiquitous risk factors, there should be more cases than we expected. It is no doubt that IPF is slowly becoming a stumbling block to keeping people healthy worldwide. IPF is associated with poor prognosis, cough, dyspnea, and diminished quality of life. Drug intervention, symptom-oriented therapies, and pulmonary transplantation are the three well-accepted clinical treatments of IPF (Raghu and Richeldi, 2017; Richeldi et al., 2017).

During the pathogenesis of IPF, alveolar epithelial cells undergo repetitive microinjuries. This damage generates the secretions of coagulants, cytokines, and fibrogenic growth factors such as transforming growth factor-beta 1 (TGF-β1) (Horowitz and Thannickal, 2006). TGF-β1 could induce differentiation of fibroblasts, myofibroblast recruitment, mesenchymal cell proliferation, and epithelial–mesenchymal transition (EMT) (Rock et al., 2011; Hung et al., 2013). The myofibroblast abnormal deposit leads to the excess synthesis of extracellular matrix (ECM) proteins such as collagen I in the lung tissue, which in turn promotes the differentiation of fibroblast to myofibroblast (Parker et al., 2014). In the signaling pathway that regulates IPF, the Smad protein family is a downstream molecule in the TGF-β1 signaling pathway. TGF-β1 promotes Smad2/3 to bind to Smad4, and then the Smad complexes are transmitted into the nucleus and regulate the expressions of target proteins related to ECM, EMT, and profibrotic mediators finally (Hu et al., 2018). Therefore, given the effects of the TGF-β1/Smad signaling pathway on collagen synthesis and EMT, blocking this pathway has become a vital therapeutic strategy in the IPF treatment.

The past 30 years have seen a proliferation of studies showing that blueberry has the best health benefits, such as anti-inflammation, reduction in oxidative stress, prevention of cardiovascular diseases, and anticancer. Bioactive components in blueberries include various kinds of anthocyanins (anthocyanidins, or phenolic aglycone, conjugated with sugar), tannins, chlorogenic acid, citric acid, arbutin, myricetin and its glycoside, flavonoids, pterostilbene, resveratrol, and so on (Chen et al., 2010; Yang and Jiang, 2010). Mounting evidences suggested that these phytochemicals, either individually or synergistically, contribute to the health promotion activity of blueberry (Skrovankova et al., 2015). Recently, a few studies have focused on the anti-fibrosis activity of blueberry. In these studies, rats were administrated orally with blueberry juice (BBJ), and it was discovered that BBJ could alleviate hepatic fibrosis or injury through reducing the expressions of NF-κB p65 and TGF-β1 (Lu et al., 2012; Wang et al., 2013; Zhan et al., 2017; Zhang B. F. et al., 2018). However, the therapeutic use of BBJ and its role in IPF have not been investigated yet. Therefore, this study is designed to evaluate the anti-fibrotic activity of BBJ using various fibroblasts *in vitro* and bleomycin-induced pulmonary fibrosis models *in vivo* to further elucidate its potential mechanism.

## 2 Materials and Methods

### 2.1 Reagents

Bleomycin (BLM) sulfate was purchased from Chengdu Synguider Technology Co., Ltd (Chengdu, China). Nintedanib was from Chengdu Giant Pharmaceutical Technology Co., Ltd (Chengdu, China). TGF-β1 was purchased from Novoprotein (Shanghai, China). Ten micrograms of TGF-β1 was added into 100 μl sterile ddH_2_O and mixed well. Then 0.1 g/L TGF-β1 solution was kept at −80°C.

### 2.2 BBJ Extraction

Fresh blueberry fruits produced from Peru were bought in the market, weighed, washed, and wiped with absorbent papers. Then the fruits were juiced by blender (Joyoung, China) and centrifuged at 4,000 rpm for 5 min. After being filtered by 0.22 μm membrane filters, the supernatant was stored separately and protected from light at −80°C. The yield rate was about 0.24 ml/g (fresh weight), and the mass concentration of BBJ is 1.01 g/ml.

### 2.3 LC/MS Analysis

The ingredients of BBJ were detected by the LC/MS system (Thermo Scientific Q Exactive). In the experiment, a CAPCELL PAK-C18 column (100 mm × 2.1 mm, 2.7 μm) was used. Mobile phase A was the solution with 0.1% formic acid. Mobile phase B was acetonitrile. The gradient was as follows: 0–2 min, 5% B; 2–5 min, from 5% to 30% B; 5–7 min, from 50% to 70% B; 7–8.1 min, from 75% to 90% B; 8.1–10 min, from 95% to 5% B. The flow rate was set as 0.3 ml/min. The injection volume of the sample was 10 μl, and the column temperature was kept at 40°C. The data were analyzed by Compound Discoverer 3.3.3.12 and matched with mzVault and mzCloud databases. Results were visualized by Thermo Xcalibur Qual Browser and Origin 2018 software. Before being injected into the column, samples and mobile phases were filtered by 0.22 μm membrane filters and degassed.

### 2.4 Total Phenols and Flavonoid Content Determination

The total phenol content of BBJ was detected according to a previous study with some modifies (Czerwiński et al., 2004). Phenol components in 1 ml BBJ diluted in 9 ml 75% ethanol were extracted in ultrasound for 30 min. The extraction was used to determine total phenols and flavonoids. Total phenols were measured at 765 nm using a Folin reagent with chlorogenic acid as a standard. Total flavonoid content was estimated by NaNO_2_-Al(NO_3_)_3_-NaOH colorimetric methods, according to previous description (Bao et al., 2015). And rutin was set as standard. These measurements were conducted in triplicate. Results were expressed as μg/g of BBJ and mean ± SD.

### 2.5 Antioxidant Ability Test

To determine the antioxidant ability of BBJ, DPPH, Fenton reactions, and ABTS experiments were performed according to related work (Bao et al., 2015). These measurements were conducted in triplicate. The half maximal inhibitory concentration (IC_50_ value) was calculated by Excel.

#### 2.5.1 DPPH Assay

For DPPH assay, 5 μl BBJ or Vitamin C was incubated with 195 μl of a 0.035 mg/ml DPPH ethanol solution for 30–40 min at room temperature. The absorbance (A_1_) was measured at 517 nm. The DPPH radical scavenging activity (C%) was expressed by the following formula:

$$\mathrm{C\%}=\left(1-\frac{A_{1}-A_{2}}{A_{0}}\right)\times 100\%$$

In the formula, *A*_2_ stands for the OD value of the mixture of BBJ or Vitamin C and ethanol solution; *A*_0_ is the absorbance of the mixture of ddH_2_O and DPPH solution.

#### 2.5.2 Fenton Reaction

For the detection of hydroxyl free radical HO scavenging ability, 7.5 mM ferrous sulfate solution, 6 mM salicylic acid ethanol solution, 0.3% H_2_O_2_, and different concentrations of BBJ or Vitamin C were added into the tubes. Each volume of these solutions was 0.25 ml. After a water bath at 37°C for 30 min, the absorbance of the 200 μl mixed solution was tested at 520 nm (*A*_1_). The hydroxyl free radical HO scavenging ability (C%) was expressed by the formula in Section 2.5.1. In the formula, *A*_2_ stands for the OD value of the mixture of 7.5 mM ferrous sulfate solution, 6 mM salicylic acid ethanol solution, ddH_2_O, and different concentrations of BBJ or Vitamin C; *A*_0_ is the absorbance of the mixture of 7.5 mM ferrous sulfate solution, 6 mM salicylic acid ethanol solution, 0.3% H_2_O_2_, and ddH_2_O.

#### 2.5.3 ABTS Assay

In ABTS assay, 7.4 mM ABTS solution and 2.6 mM potassium persulfate aqueous solution were mixed in equal volume and reacted for 12–16 h, avoiding exposure to light, as ABTS stock solution. The ABTS stock solution was diluted by 20 mM sodium acetate aqueous solution for work solution. Ten microliters of BBJ or Vitamin C was added into 195 μl ABTS work solution. After incubation for 5 min, avoiding exposure to light, the absorbance of mixed solution was determined at 734 nm (*A*_1_). The ABTS scavenging ability was calculated by the formula in Section 2.5.1. In the formula, *A*_2_ stands for the OD value of the mixture of 10 μl different doses of BBJ or Vitamin C and 195 μl sodium acetate solution; *A*_0_ is the absorbance of the mixture of 10 μl ddH_2_O and 195 μl ABTS work solution.

### 2.6 Rat Primary Lung Fibroblast (RPLF) Isolation and Cell Culture

RPLFs were isolated from 7- to 8-week-old specific pathogen-free (SPF) male Wister rats with lung fibrosis (permit number: 2018091812). The weight of each rat was about 300 g. After being anesthetized by intraperitoneal injection of 1 ml 10% chloral hydrate solution, the rats were injected with 4 mg/kg body weight BLM saline solution through intratracheal infusion. A week later, the rats were sacrificed. The lung tissues were removed and washed with sterilized Hank’s solution. After the fascia were cut off, the lung tissues were minced and resuspended in 10 ml 0.25% trypsin for 40 min at 37°C. The suspension was then filtered by 70 μm nylon mesh and centrifuged at 1,500 rpm for 5 min. The precipitates were resuspended with 10 ml DMEM-F12 medium and centrifuged at 500 rpm for 5 min. The supernatant was centrifuged for 5 min at 1,500 rpm. The supernatant was discarded, and the cells were precipitated and cultured in fresh DMEM-F12 medium with 1% antibiotics (penicillin and streptomycin, MP Biomedical LLC) and 10% heat-inactivated fetal bovine serum (GIBCO, NY) in a culture dish at 37°C in a 5% CO_2_ humidified incubator.

HPF (human pulmonary fibroblast, from ATCC) cells were cultured by DMEM with 20% FBS and 1% antibiotics. A549 (human lung carcinoma epithelial cells, from ATCC), NIH/3T3 (mouse embryo fibroblasts, from ATCC), and LO2 (human liver cells, from ATCC) were cultured by DMEM with 10% FBS and 1% antibiotics.

### 2.7 MTT Experiment, Apoptosis Assay, Reactive Oxygen Species (ROS), and Mitochondrial Membrane Potential (ΔΨm) Detections

Cells were seeded in 96-well plates with appropriate number. Overnight, various doses (0–100 μl/ml) of BBJ and 200 μl/ml normal saline (NS) or nintedanib (NTB) were added. After 24, 48, and 72 h, cells were incubated with MTT for 3 h, the supernatant was discarded, and 150 μl/well DMSO was added. The OD value was detected at the length of 570 nm.

Cells were seeded in six-well plates. After adhering to the plates, cells were treated with BBJ and/or TGF-β1 for 24 h. For apoptosis assay, apoptotic cells were tested by an apoptosis kit (KeyGen Biotech, Nanjing, China) and analyzed by a NovoCyte™ flow cytometer (ACEA Bioscience, Inc., CA, USA). For intracellular ROS and ΔΨm detections, DCFH-DA and Rh123 were applied as related dyes and determined using flow cytometry.

### 2.8 Wound-Healing Assay and Cell Morphology

A549 cells were seeded into a six-well plate. When the cells grew to about a density of 85%, cells were cultured with the DMEM without FBS for 6 h. Then, wounds were made by a sterile 100 μl pipette tip. We changed the medium into DMEM with 3% FBS, and cells were treated with 0 or 5 ng/ml TGF-β1. After 1 h, cells were treated with 0 or 50 μl/ml BBJ for 24 h, and these four wells were treated with 0 μl/ml BBJ and 0 ng/ml TGF-β1, 50 μl/ml BBJ and 0 ng/ml TGF-β1, 0 μl/ml BBJ and 5 ng/ml TGF-β1, and 50 μl/ml BBJ and 5 ng/ml TGF-β1, separately. The scratches were photographed at 0 and 24 h. The areas of scratches were analyzed by ImageJ software. Wound closure rate was expressed by the ratio of the difference between the wound area at 0 and 24–0 h. The cell morphology was observed by a microscope when A549 cells were treated with BBJ and TGF-β1 for 24 h.

### 2.9 Immunofluorescence Assay

A549, NIH/3T3, and HPF were seeded into 24-well plates precoated with sterile cell slides. Overnight and after starvation for 6 h, these cells were treated with BBJ and TGF-β1 as expressed in wound-healing assay for 24 h. Cells were washed with cold PBS, fixed with 4% paraformaldehyde for 15 min, permeabilized with 0.5% Triton-100 solution for 20 min, and blocked with PBS containing 0.05% Triton-100 and 5% bovine serum albumin. Next, cells were incubated with α-SMA (1:150, Abcam, Cambridge, MA) and E-cadherin (1:200, Abcam, Cambridge, MA) for 18 h at 4°C. Then cells were washed with PBST and incubated with FITC or PE-conjugated goat anti-rabbit/mouse antibody (1:200, Alexa Fluor 488 or 647, Life Technologies, Waltham, MA) for 1.5 h at room temperature in the dark. The nuclei were stained by DAPI (Roche Molecular Biochemicals, Inc., Pleasanton, CA) for 10 min, protected from light. The fluorescence was detected by a confocal microscope (Zeiss LSM 880, Germany).

### 2.10 Animal Study

All animal experiments were approved and performed in compliance with the Animal Care and Use Committee of Sichuan University in China. To explore the preventive activity of BBJ, male C57BL/6 mice (6–8 weeks old) were obtained from Beijing HFK Bioscience Co., Ltd., and bred in an SPF condition. After being maintained for 1 week, each anesthetized mouse was injected with 60 μl saline that contained BLM sulfate (approximately 2 mg/kg body weight) by intratracheal instillation, while the sham group was injected with normal saline at the same volume. These mice were divided into four groups: the sham group, vehicle group, BBJ-L (5 ml/kg body weight) group, and BBJ-H (10 ml/kg body weight) group. The sham and vehicle groups were treated with normal saline at a dose of 10 ml/kg body weight. Saline and BBJ were administrated by gavage every day for 4 weeks. The mice were sacrificed at the end of the study.

As for the treatment effect of BBJ on IPF, male C57BL/6 mice (6–8 weeks old) were obtained from Beijing HFK Bioscience Co., Ltd., and bred in an SPF condition. When the weights of mice are over 20 g, mice were anesthetized and injected with 100 μl saline that contained BLM sulfate (approximately 1 mg/kg body weight) by intratracheal instillation. The sham group was injected with normal saline at the same volume. After 1 week, these four groups, sham group (10 ml saline/kg body weight), vehicle group (10 ml saline/kg body weight), BBJ group (10 ml BBJ/kg body weight), and NTB group (30 mg/kg body weight, resolved in the solvent in which the proportion of DMSO, PEG400 and saline was 0.5:3.5:6), started to be administrated every day by gavage. This treatment lasted for 3 weeks, and lung tissues from sacrificed mice were kept.

### 2.11 Hematoxylin and Eosin (H&E), Masson, and Immunohistochemistry (IHC) Staining

The left lung tissues, liver, heart, spleen, and kidney were kept in 4% paraformaldehyde. Next, the lung tissues were rinsed with flowing water, dehydrated, embedded with paraffin, and cut into discontinuous sections at a thickness of 3 μm. Through dewaxing and washing, these sections were stained with H&E, and lung sections were stained with Masson’s trichrome by standard protocols. For IHC assay, sections were incubated with primary antibodies (α-SMA, collagen I, p-Smad3, and p-Stat3). All pathological sections were scanned by a Pannoramic MIDI II 3DHISTECH digital pathology system, and the pictures were analyzed with the CaseViewer software. We quantified the pulmonary fibrosis according to H&E results by the method of modified Ashcroft scale (Hübner et al., 2008). Collagen volume fraction and IHC score were analyzed by ImageJ software.

### 2.12 IL-10 and IL-17A Contents in Serum

The detection of IL-10 and IL-17 contents in serum was followed with protocols in a Th1/Th2/Th17 CBA kit (BD Pharmingen, USA).

### 2.13 Hydroxyproline Content Detection

Right lung tissues were kept at −80°C. About 30 mg lung tissues were weighed and hydrolyzed by alkaline. The contents of hydroxyproline were measured by the hydroxyproline assay kit (A030-2-1, Nanjing Jiancheng Bioengineering Institute, China).

### 2.14 Western Blotting

Cells or lung tissues were lysed with RIPA buffer containing protease and phosphatase inhibitor cocktail (Selleck Chemicals, Houston, TX, USA). Protein concentrations were detected by the Bradford method. Western blotting was performed as before (Li et al., 2019). The primary antibodies α-SMA, collagen-I, E-cadherin, and vimentin were purchased from Abcam (Cambridge, MA); Stat3, p-Stat3, Smad2/3, and p-Smad2/3 were from Cell Signaling Technology Company; β-actin were bought from ZSJQ-BIO (Beijing, China) and were involved in western blotting.

### 2.15 Statistical Analysis

Results were expressed as mean ± SD. We analyzed numeric data for statistical significance using the independent-sample *t*-test. A *p*-value <0.05 was considered as significant.

## 3 Results

### 3.1 Ingredients, Total Phenol, and Flavonoid Contents in BBJ and Antioxidant Ability

The yield rate of BBJ was about 24% (ml juice/100 g fresh fruit). To figure out the components of BBJ, the LC/MS system was used. After being matched with the mzVault and mzCloud database, 30 possible substances, whose suitability was both over 80 or more than 90 in one database, were displayed in Supplementary Table S1. And the total ion chromatography result in determination is shown in Supplementary Figure S1. We found carbohydrates, amino acids, liquids, vitamins, and phytochemicals including phenols in BBJ.

According to this result, we used chlorogenic acid as a standard to determine the total phenols in BBJ. As shown in Table 1, the contents of total phenols and flavonoids in BBJ were 1,219.15 and 603.89 μg/g, respectively.

**TABLE 1:** The total phenols and flavonoid contents in BBJ.

	Standard curve	*R* ^2^	Standard	Content (μg/g)
Total phenols	Y = 0.0022 × x − 0.0032	0.9953	chlorogenic acid	1,219.15 ± 75.26
Total flavonoids	Y = 0.0010 × x + 0.0035	0.9983	rutin	603.89 ± 29.21

Total phenol content was determined by Folin reagent, using chlorogenic acid as standard; total flavonoids in BBJ were detected by NaNO_2_-Al(NO_3_)_3_-NaOH colorimetric methods with rutin. Results were shown as mean ± SD from replicate determinations.

According to Table 2, IC_50_ values of BBJ in DPPH, Fenton, and ABTS tests were 46.69, 7.24, and 4.01 g/100 ml, respectively. And to eliminate 50% of free radicals in these three assays, 17.21, 20.58, and 658.29 mg/100 ml of Vitamin C were needed, which meant that BBJ’s antioxidant ability was weaker than Vitamin C. However, plenty of water in BBJ should be taken into consideration when evaluating the antioxidant ability. Additionally, according to published research, the ABTS method is more suitable than DPPH assay to detect and evaluate the antioxidant activity of pigments and hydrophilic antioxidants in blueberries (Mishra et al., 2012; Li et al., 2017).

**TABLE 2:** The antioxidant activity (IC_50_ values) of BBJ and Vitamin C.

	DPPH assay	Fenton reaction	ABTS assay
BBJ (g/100 ml)	46.69 ± 0.33	7.24 ± 0.33	4.01 ± 0.11
Vitamin C (mg/100 ml)	17.21 ± 0.65	20.58 ± 0.69	658.29 ± 3.06

To verify the antioxidant ability of BBJ, Fenton reaction, DPPH, and ABTS assays were applied. Vitamin C was set as the positive control. Half maximal inhibitory concentrations were calculated in Excel. Results were shown as mean ± SD from replicate determinations.

### 3.2 BBJ-Induced Cell Death and Apoptosis in NIH/3T3 and HPF

**FIGURE 1:**
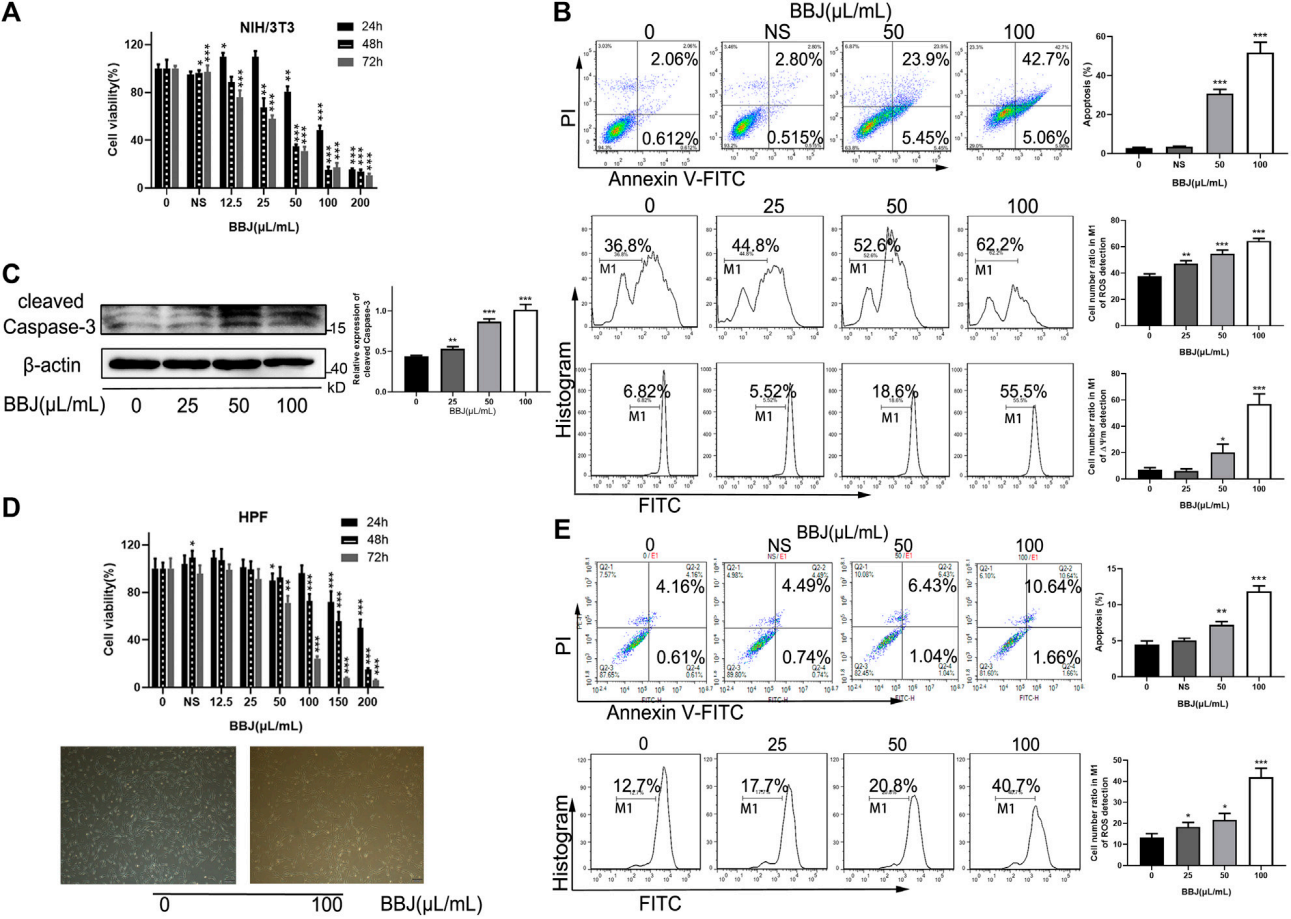
BBJ induced apoptosis in NIH/3T3 and HPF. **(A)** NIH/3T3 was treated with BBJ or NS for 24, 48, and 72 h. The cell viability was disclosed by MTT assay. **(B)** Apoptotic rate, ROS, and ΔΨm of NIH/3T3 treated with BBJ for 24 h were analyzed and expressed as bar histograms. **(C)** Expression of cleaved caspase-3 in NIH/3T3 was detected through a western blot assay and calculated by ImageJ after incubation with BBJ for 24 h. **(D)** Under treatment with various doses of BBJ for 24 h, the viability of HPF was determined by MTT tests, and cell morphology was observed. **(E)** HPF was dealt with BBJ for 24 h, and apoptosis and ROS were analyzed by flow cytometry. *p < 0.05; **p < 0.01; ***p < 0.001 compared with the control group.

We first investigated the antifibrotic activity of BBJ treatment on cell proliferation using NIH/3T3 and HPF. As shown in Figures 1A,D, we found that BBJ treatment significantly inhibited cell proliferation in a time- and dose-dependent manner. When intervened with 50 μl/ml BBJ for 24 h, cell viability rates were between 70% and 90% (Figures 1A,D). Additionally, compared with the vehicle group, an extra 27.68% apoptotic cells and 15.80% and 11.78% loss of ROS and ΔΨm, respectively, were induced by 50 μl/ml BBJ in NIH/3T3, as shown in Figure 1B. BBJ stimulated apoptosis in NIH/3T3 *via* the upregulated cleaved caspase-3 (Figure 1C). In HPF, as revealed in Figure 1E, an additional 2.80% of apoptotic rate and 8.10% of intracellular ROS loss were induced by 24 h treatment of 50 μl/ml BBJ, compared with the vehicle group. To sum up, these results suggested that the BBJ have potential antioxidant ability and can trigger cell death in NIH/3T3 and HPF *in vitro*.

### 3.3 BBJ Suppressed Differentiation in NIH/3T3 and HPF Through Inhibiting TGF-β1/Smad2/3 Signaling

**FIGURE 2:**
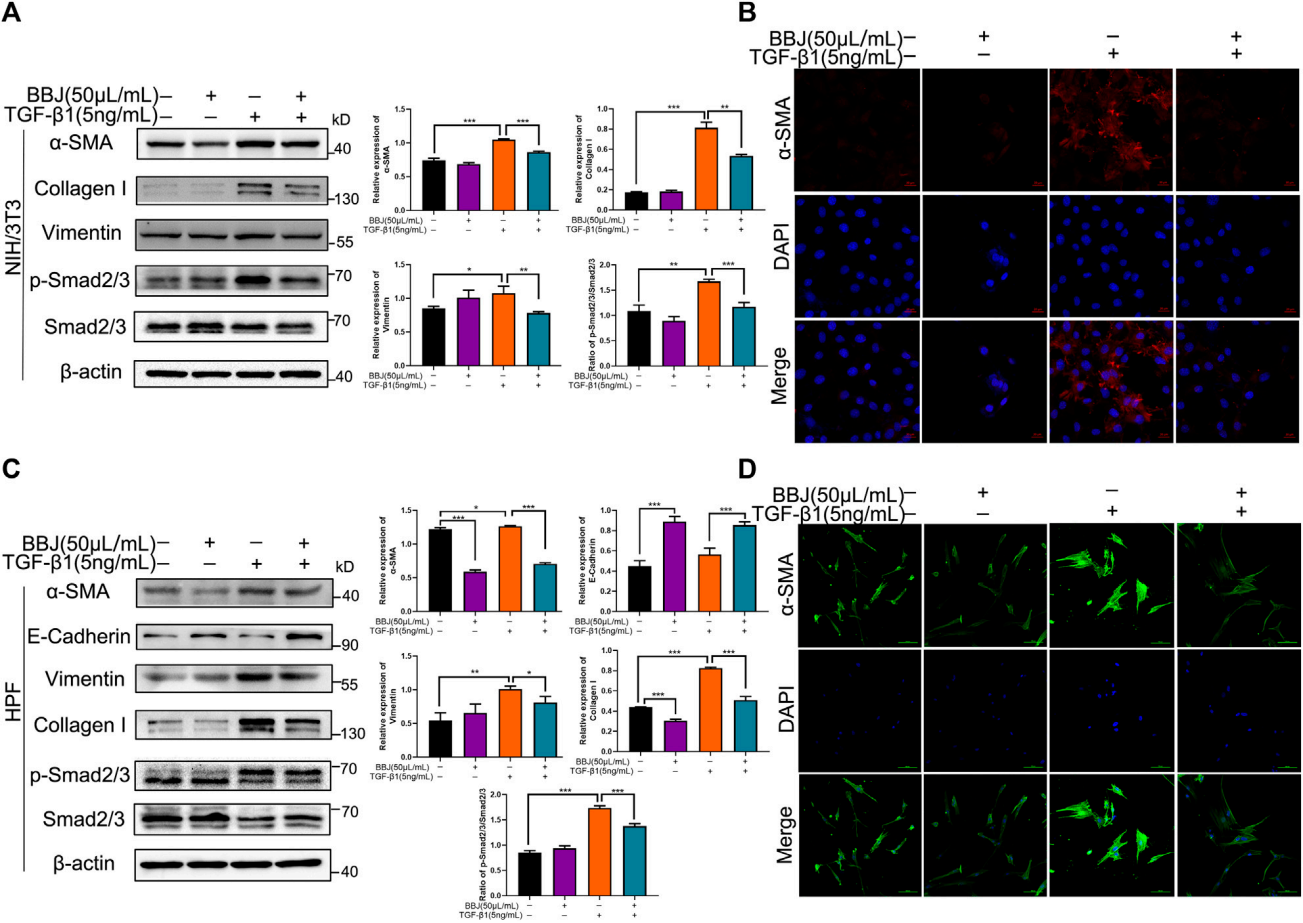
BBJ restrained activation and differentiation of NIH/3T3 and HPF induced by TGF-β1. NIH/3T3 or HPF was planted, starved for 6 h, stimulated by 5 ng/ml TGF-β1 for 1 h, and then incubated with 50 μl/ml BBJ for an extra 24 h. **(A)** Expressions of α-SMA, vimentin, collagen-I, Smad2/3, p-Smad2/3, and β-actin in NIH/3T3 were presented by western blotting. **(B)** The expression of α-SMA in NIH/3T3 was visualized by a confocal microscope (×40). **(C)** Expressions of α-SMA, E-cadherin, vimentin, collagen-I, Smad2/3, p-Smad2/3, and β-actin in HPF were tested by western blotting. **(D)** The expression of α-SMA in HPF was visualized by a confocal microscope (×40). *p < 0.05; **p < 0.01; ***p < 0.001 versus the control group or the TGF-β1 group.

Numerous studies have shown that TGF-β is an important cytokine in the development of fibrosis and recruits downstream Smad2/3 proteins. To uncover the potential mechanism of BBJ’s anti-lung fibrosis in fibroblasts, TGF-β1 was applied to lead fibroblast activation. In NIH/3T3 and HPF cells, TGF-β1 induced upregulation of α-SMA, collagen-I, and vimentin. In other words, TGF-β1 caused fibroblast differentiation successfully. However, the addition of BBJ could suppress this increase, as shown in the results of western blot (Figures 2A,C). And immunofluorescence results, as exhibited in Figures 2B,D, verified that BBJ restrained the upregulation of α-SMA induced by TGF-β1. More importantly, BBJ decreased the high ratio of p-Smad2/3 to Smad2/3 expression caused by TGF-β1 in NIH/3T3 and HPF. In conclusion, these changes indicated that BBJ could inhibit lung fibrosis through the TGF-β1/Smad2/3 pathway.

### 3.4 BBJ Had an Inhibitory Effect in RPLF

**FIGURE 3:**
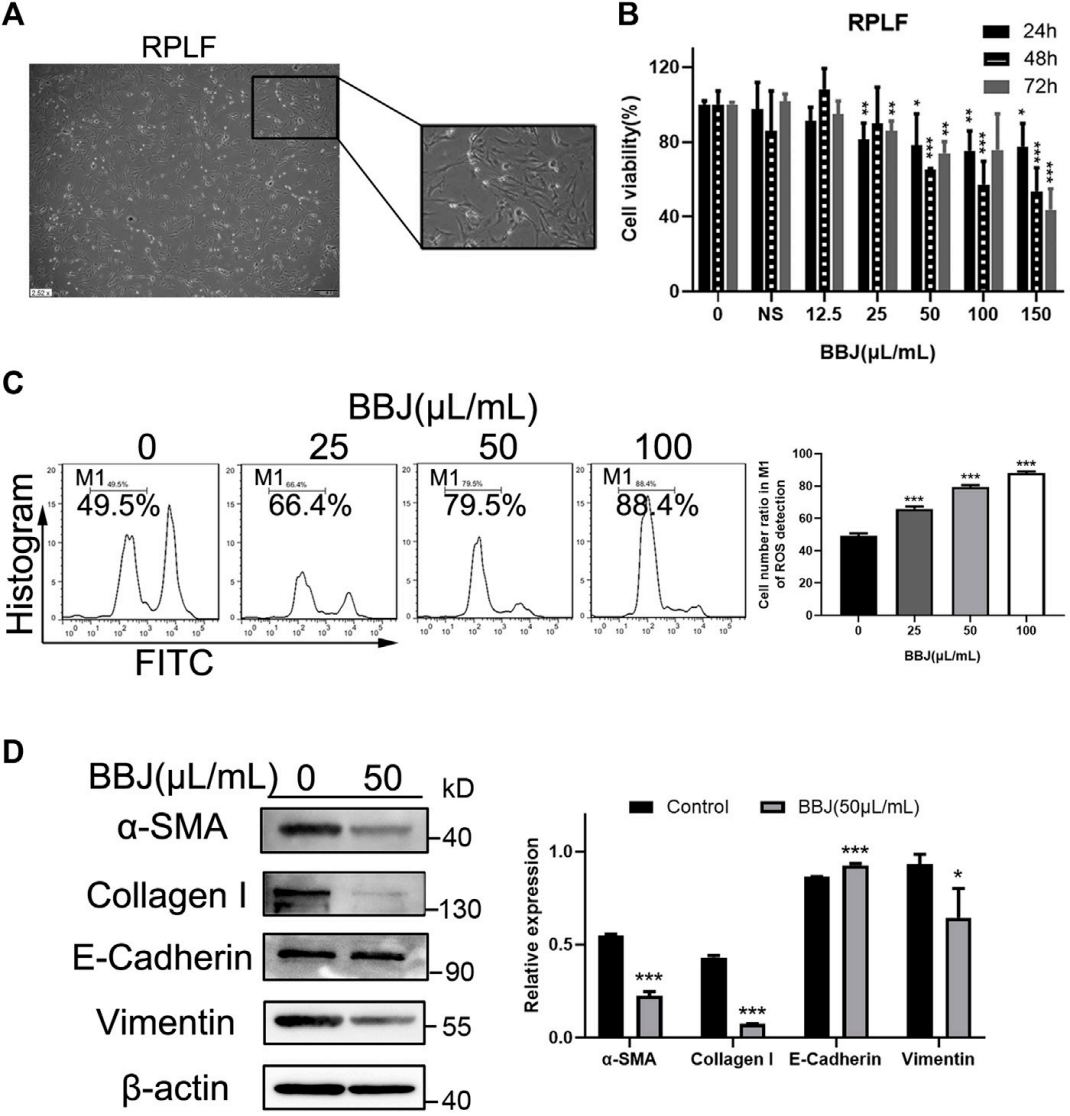
The inhibitory effect of BBJ in RPLF. **(A)** Cell morphology of RPLF was photographed by an inverted microscope (×10). **(B)** RPLFs were treated with BBJ or normal saline for 24, 48, and 72 h. Cell viability was detected by MTT tests. **(C)** ROS in RPLF was analyzed after 24 h treatment of BBJ or NS. **(D)** Expressions of α-SMA, E-cadherin, vimentin, collagen-I, and β-actin in RPLF were determined by western blot assay. Protein expressions were treated statistically by ImageJ. *p < 0.05; **p < 0.01; ***p < 0.001 versus the control group.

To further illustrate BBJ’s disincentive function in fibroblasts, RPLF cells were extracted from the pulmonary fibrosis model, which was built by BLM tracheal infusion in male rats. RPLFs were considered as active fibroblasts. The cell morphology of RPLF was observed under an inverted microscope, presenting a slender fusiform with two to four antennae (Figure 3A). When RPLFs were treated with BBJ, their cell viability dropped, as shown in Figure 3B. The IC_50_ value of BBJ at 24 h in RPLF was over 200 μl/ml, while that in NIH/3T3 was 95.66 μl/ml and that in HPF was 145.47 μl/ml. As shown in Figure 3C, BBJ apparently gave rise to ROS loss in RPLF. BBJ exerted the ability to degrade expressions of α-SMA, collagen-I, and vimentin as well as increase E-cadherin slightly at 50 μl/ml (Figure 3D). In summary, these outcomes in RPLF further confirmed that BBJ has antifibrotic effects *in vitro*.

### 3.5 BBJ Could Restrain the EMT Process in A549 Cells

**FIGURE 4:**
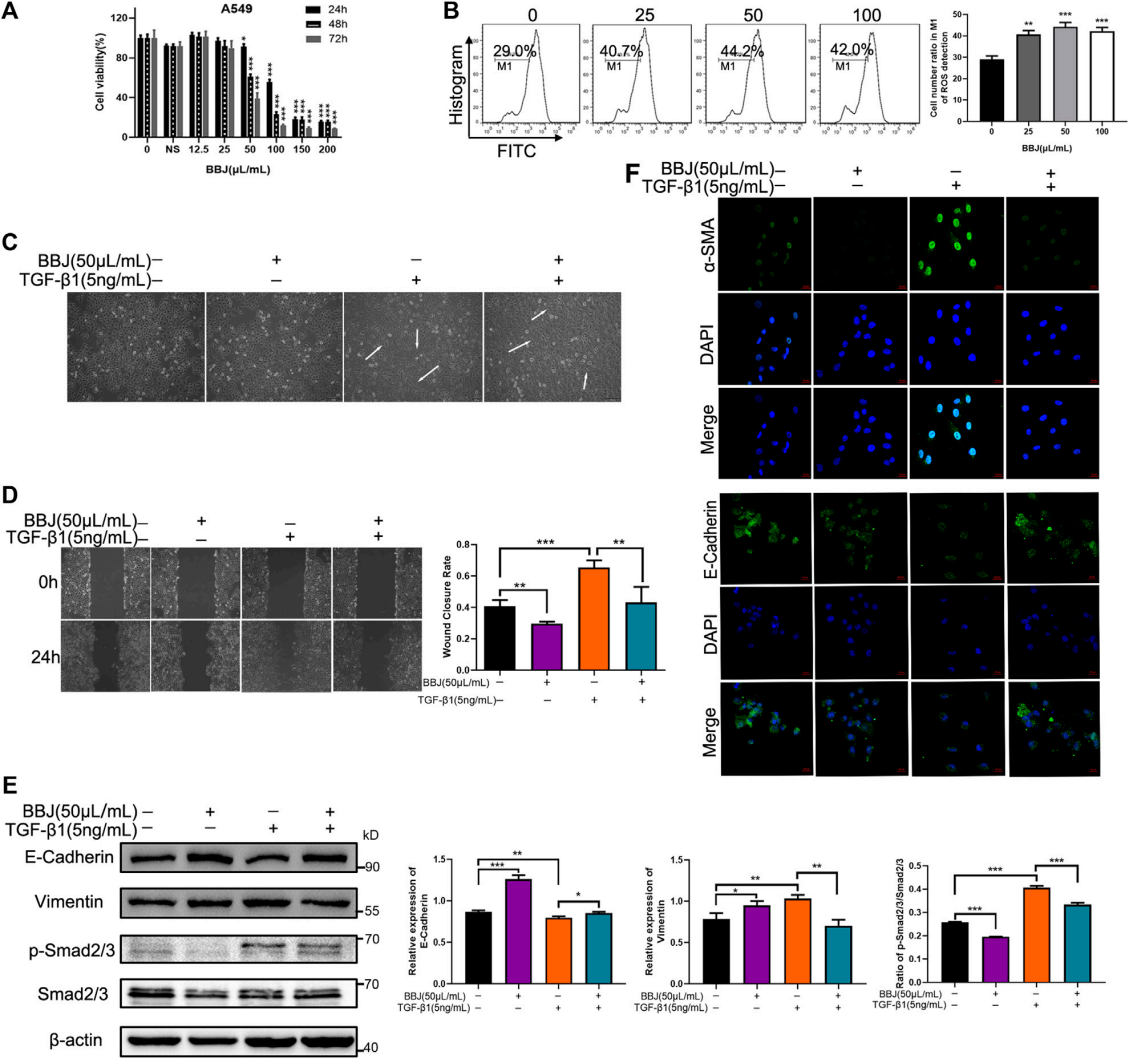
BBJ could reverse EMT progress in A549. **(A)** The cytotoxicity of BBJ on A549 was determined by an MTT test. A549 cells were treated with different doses of BBJ and 200 μl/ml normal saline for 24, 48, and 72 h. Data were presented as mean ± SD from three experiments. **(B)** ROS of A549 treated with BBJ for 24 h were detected by flow cytometry and counted. **(C)** After starvation for 6 h with serum-free medium, fresh medium with 5 ng/ml TGF-β1 was replaced. One hour later, 50 μl/ml BBJ was added. After 24 h, cell morphology of A549 was observed by a microscope (×10). **(D)** A549 cells were seeded in six-well plates for 24 h and starved for 6 h with serum-free medium. Then, a scratch was made by 100 μl pipette tips, and fresh medium with 5 ng/ml TGF-β1 was added. One hour later, 50 μl/ml BBJ was added. The scratches were photographed at 0 and 24 h by an inverted microscope (×10). The scratch areas were treated statistically by ImageJ. **(E)** After incubation with 5 ng/ml TGF-β1 for 1 h and 50 μl/ml BBJ for an extra 24 h, expressions of E-cadherin, vimentin, p-Smad2/3, Smad2/3, and β-actin in A549 were analyzed by western blotting. **(F)** After stimulation by TGF-β1 for 1 h and treatment with BBJ for 24 h, expressions of α-SMA and E-cadherin were visualized by a confocal fluorescence microscope (×40). *p < 0.05; **p < 0.01; ***p < 0.001 versus the control group or TGF-β1 group.

EMT plays an important role in many respiratory diseases, especially in fibrosis. To make explicit the antifibrotic ability of BBJ, A549 cells were used as a model of EMT. Firstly, MTT assay was utilized to determine the cytotoxicity of BBJ. The IC_50_ value at 24 h was 107.63 μl/ml (Figure 4A). At a dosage of 50 μl/ml, A549 cell viability was around 90%. Moreover, 50 μl/ml of BBJ had just induced 15.20% more ROS loss than the vehicle group (Figure 4B). Hence, in the following tests, A549 cells were treated with 50 μl/ml of BBJ. TGF-β1 induced EMT in A549, including changes of cell morphology, enhanced cell migration, and altered related proteins’ expressions. Firstly, we observed the significant increased proportion of elongated cells induced by TGF-β1, which was reversed by BBJ (Figure 4C and Supplementary Figure S2). As shown in Figure 4D, 24 h treatment of 50 μl/ml BBJ could inhibit A549 cell migration stimulated by 5 ng/ml of TGF-β1 apparently. In the meantime, western blotting and immunofluorescence assays were utilized to elucidate mechanisms by which BBJ restrained the EMT process in A549. α-SMA, E-cadherin, and vimentin were involved in the EMT process. TGF-β1 could induce upregulation of α-SMA, p-Smad2/3, and vimentin as well as downregulation of E-cadherin. BBJ reversed these changes significantly, which is expressed in Figures 4E,F.

### 3.6 BBJ Could Suppress Formation and Development of Lung Fibrosis *In Vivo*

**FIGURE 5:**
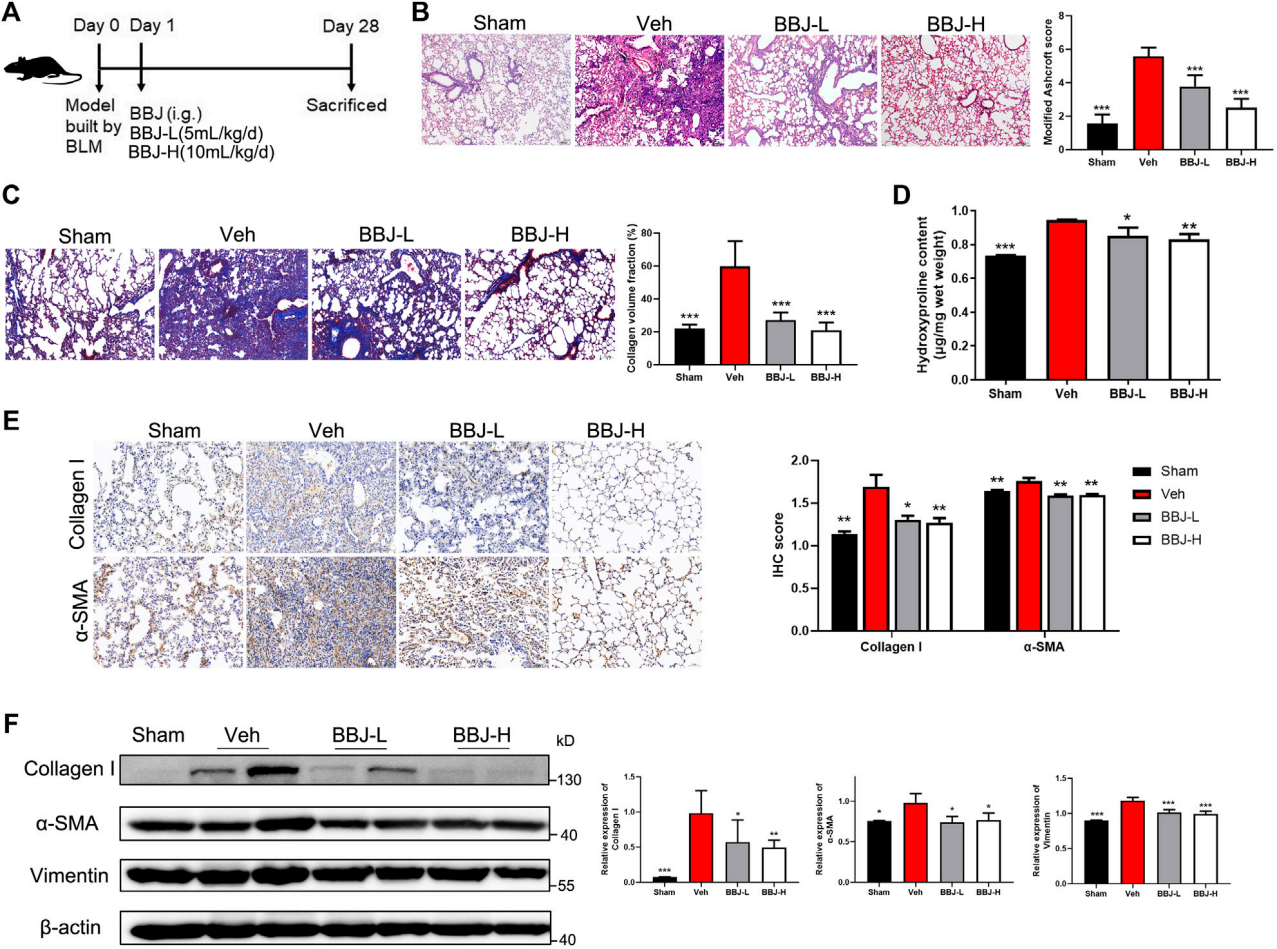
The BBJ could prevent fibrosis formation induced by BLM in mice. **(A)** Arrangement of animal study in the prevention model. The fibrosis model was built by tracheal injection of BLM on day 0. Next day, vehicle and BBJ-fed groups were administrated by saline and BBJ for 4 weeks, respectively. **(B)** Lung sections of each group were stained with H&E (×10). Modified Ashcroft scores were applied to evaluate the degree of pulmonary fibrosis. **(C)** Lung sections of each group were stained by Masson trichrome (×10). Collagen volume fractions were analyzed from the results of Masson-stained lung sections by ImageJ. **(D)** Hydroxyproline contents were tested by the hydroxyproline assay kit. **(E)** The expressions of collagen-I and α-SMA in lung sections were detected by IHC staining, and these proteins’ expressions were calculated by ImageJ. **(F)** Expressions of collagen-I, α-SMA, vimentin, and β-actin in lung tissues of each group were analyzed by western blotting. ImageJ software was used to calculate these proteins’ expressions. *p < 0.05; **p < 0.01; ***p < 0.001 compared with the vehicle group.

To determine the antifibrotic ability of BBJ, pulmonary fibrosis models were built through administration of BLM (2 mg/kg for prevention model and 1 mg/kg for treatment model). The sham group was injected with saline. As shown in Figure 5A, mice were intragastrically administrated with BBJ or saline every day from day 1 in the prevention model. After 4 weeks, mice were sacrificed, and lung tissues were harvested for histopathology, immunoblot, and hydroxyproline content analyses. As explicated in Figures 5B,C, H&E- and Masson-stained results were present. According to the H&E-stained lung sections in the vehicle group, there were variable alveolar septa and large contiguous fibrotic masses (about 40% of the microscopic field). Furthermore, the lung architecture was severely damaged, and part of it was not preserved. After intervention with BBJ, the damage of the lung tissue was reduced. In Masson’s trichrome results (Figure 5C), collagen fibers were dyed blue. There was no doubt that BBJ could reduce collagen fibers compared to the vehicle group, and the lung sections of the high-dose group tended to be normal. Besides, in the BBJ group, contents of hydroxyproline in the lung tissue were lower than those in the vehicle group, which was treated with saline, as shown in Figure 5D. Moreover, contents of IL-10 and IL-17A in serum, which are involved in idiopathic pulmonary fibrosis, were lower in the BBJ group than in the vehicle group, which was explicated in Supplementary Figure 3A. To explore the underlying mechanism by which BBJ prevents lung fibrosis *in vivo*, α-SMA and collagen-I in lung tissues were determined by IHC and western blotting. β-Actin served as a reference. In Figure 5E,F, the expressions of α-SMA, collagen-I, and vimentin were upregulated evidently in the vehicle group. In contrast with the vehicle group, the BBJ-fed group showed lower expressions of these proteins.

**FIGURE 6:**
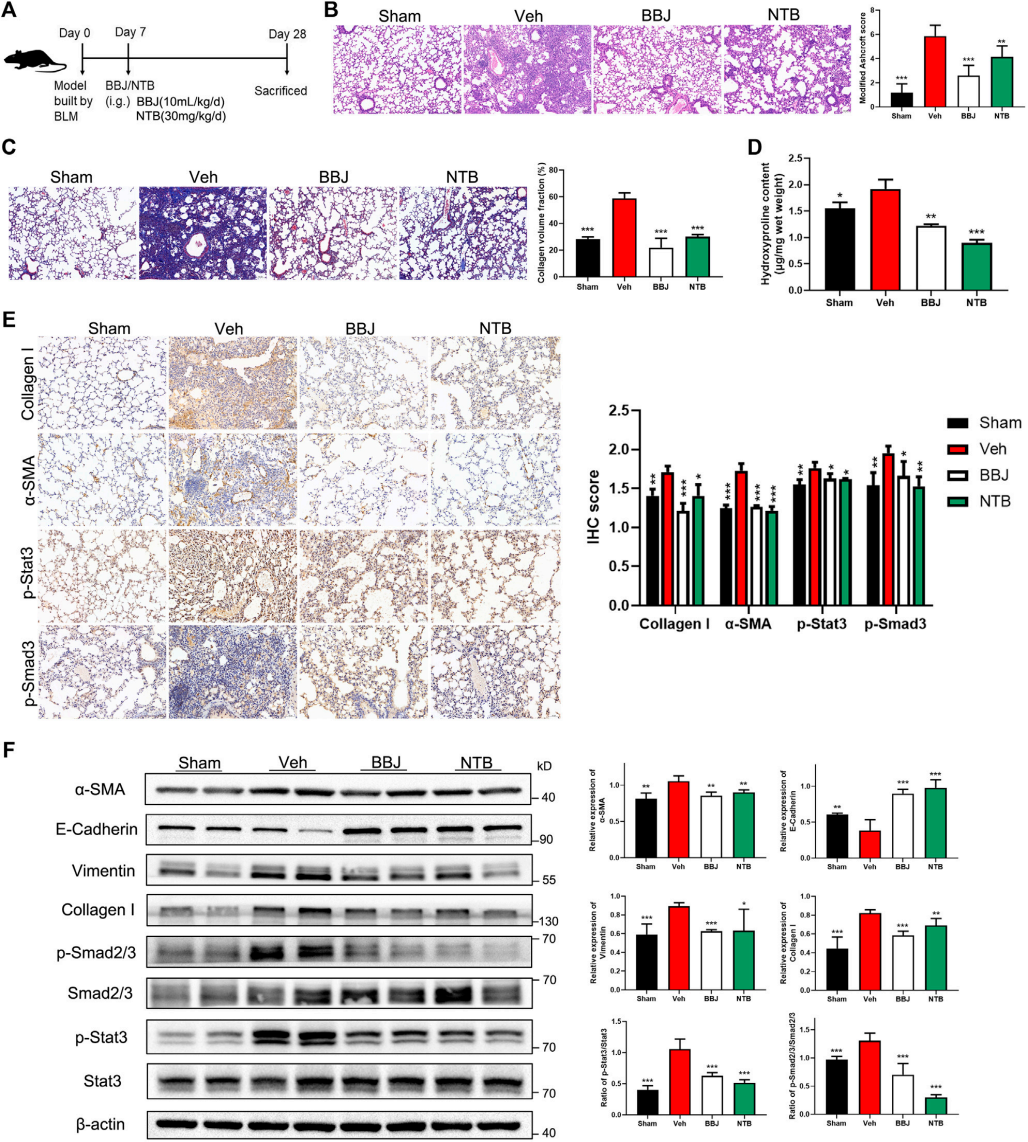
BBJ could alleviate pulmonary fibrosis caused by BLM. **(A)** Arrangement of animal study in the treatment model. The fibrosis model was built by tracheal injection of BLM on day 0. On day 7, vehicle, BBJ-fed, and NTB-fed groups were administrated with saline, BBJ, and NTB for 3 weeks, respectively. **(B)** Lung sections of each group were stained with H&E (×10). Modified Ashcroft scores were applied to evaluate the degree of pulmonary fibrosis. **(C)** Lung sections of each group were stained with Masson trichrome (×10). Collagen volume fractions were analyzed from the results of Masson-stained lung sections by ImageJ. **(D)** Hydroxyproline contents were tested by the hydroxyproline assay kit. **(E)** The expressions of collagen-I, α-SMA, p-Stat3, and p-Smad3 in lung sections were detected by IHC staining, and these proteins’ expressions were calculated by ImageJ. **(F)** Expressions of α-SMA, E-cadherin, vimentin, collagen-I, Smad2/3, p-Smad2/3, Stat3, p-Stat3, and β-actin in lung tissues of each group were analyzed by western blotting. ImageJ software was used to calculate these proteins’ expressions. *, *p* < 0.05; **, *p* < 0.01; ***, *p* < 0.001 compared with the vehicle group.

In the treatment model, nintedanib, which was reported to target tyrosine kinases, was used as positive control to verify the inhibitory effect of BBJ on IPF (Liu et al., 2021). Administrations of BBJ or NTB started from 7 days after building the lung fibrosis model and lasted 3 weeks, as displayed in Figure 6A. BBJ reduced the fibrotic degree and collagen fibers’ deposition in lung tissues, and the results coincided with those in the prevention model. Furthermore, the pulmonary fibrotic degree and deposition of collagen fibers were reduced in the BBJ and NTB groups, as shown in Figures 6B,C. We also discovered that the level of hydroxyproline, one of the main amino acids that make up collagen fibers, was decreased in the BBJ and NTB groups (Figure 6D). Similar with the prevention model, BBJ could reduce the contents of IL-10 and IL-17A in serum. Only IL-10 levels dropped in the NTB group, as explicated in Supplementary Figure 3B. The IHC and western blot results revealed that BLM increased the expressions of α-SMA, collagen-I, vimentin, p-Smad2/3, and p-Stat3 and reduced E-cadherin. Both BBJ and NTB treatment reversed these changes induced by BLM in mice (Figures 6E,F). Therefore, in summary, animal studies proved that BBJ could prevent and restrain pulmonary fibrosis *in vivo*.

## 4 Discussion

IPF is considered as a progressive respiratory disorder, ultimately causing death within about 3 years after diagnosis (Tzilas et al., 2017). There are only two drugs, nintedanib and pirfenidone, that received regulatory approval from FDA to treat IPF (Karimi-Shah and Chowdhury, 2015). However, the 5-year survival rate still remains less than 50% (Huang et al., 2015). Blueberry is well known as a significant source of nutrition, containing vitamins, fibers, and other phytochemicals with pharmaceutical interest (Miller et al., 2019). The blueberry used in the present study was from Peru, where blueberry production is dominant. Blueberries are rich in anthocyanins, polyphenols, and flavonoids. We detected 30 potential chemicals in BBJ by the method of LC/MS, which is consistent with previous studies (Chen et al., 2010; Yang and Jiang, 2010). Importantly, trehalose, quercetin, rutin, ferulic acid, abscisic acid, and trolox were reported to relieve fibrosis by inhibiting TGF-β (Galicia-Moreno et al., 2008; Bruzzone et al., 2012; Pan et al., 2014; Mu et al., 2018; Liu et al., 2019; Miyake et al., 2020). These phytochemicals were all found in BBJ and may have contributed to the anti-lung fibrosis function of BBJ. Lyophilized BBJ is reported to scavenge superoxide radicals with an IC_50_ value of 7 μg/ml and DPPH radicals with an IC_50_ value of 99 μg/ml (Cásedas et al., 2017). The many biochemicals, especially phenols, may contribute to the antioxidant property of BBJ. In the present study, we found that BBJ reduced ROS production in NIH/3T3, HPF, RPLF, and A549, which proved the antioxidant activity of BBJ. ROS is one of the indicators of the intracellular homeostasis. Accumulation of intracellular ROS could lead to cell death through ferroptosis and the mitochondrial-mediated apoptosis pathway (Li et al., 2016a; Li et al., 2020). Of note, cell death caused by phytochemicals and plant extracts is often accompanied by ROS reduction (Li et al., 2016b; Li et al., 2019). One explanation was that a decreased ROS level may induce changes in cell-cycle regulatory proteins (Qin et al., 2011). Regardless of ROS accumulation increase or decrease, the balance would be destroyed and lead to changes of cellular state, including cell death. Interestingly, there is a growing body of literature that recognizes the health benefits of blueberry, especially antifibrosis activity in rats (Chen et al., 2010; Zhan et al., 2017). Researchers manifested that BBJ could alleviate CCl_4_-induced hepatic fibrosis significantly, which was associated with reducing collagen content and α-SMA expression as well as enhancing the antioxidant capability of the liver (Wang et al., 2013). The above evidence suggested that BBJ possesses a potential antifibrosis function.

In IPF, the TGF-β1/Smad signaling pathway is the major mediator. TGF-β1-stimulated phosphorylated Smad2/3 translocates into the nucleus, regulates target gene expressions, and further participates in EMT, fibroblast proliferation, and myofibroblast differentiation, which promotes the pathogenesis of IPF (Lee et al., 2014; Shen et al., 2021). EMT, collagen deposition, and remodeling of the pulmonary interstitium are the dominant features in the pathological changes of IPF (Richeldi et al., 2017; Zhihui Zhang et al., 2018). When alveolar epithelial cells (ACEs) were attacked continuously, inflammatory repair would induce overdeposition of the extracellular matrix, which results in an abnormal lung architecture. In the EMT process, the secretion of TGF-β1 activates this pathology, which also accelerates lung fibrosis. Recently, A549 cells have served as a model of Type II-like ACEs to explore the mechanism of EMT (Foster et al., 1998; Zhang et al., 2019). BBJ induced cell death and downregulated ROS in A549. In the process of EMT, the morphology of A549 underwent a transformation from oval to fusiform once activated by TGF-β1. Meanwhile, the epithelial markers, such as E-cadherin, were downregulated while the mesenchymal markers, including α-SMA and vimentin, were upregulated. Few mesenchymal cells establish tight connections with neighboring cells, and thus, EMT promotes cell metastasis. In the present study, BBJ was proven to inhibit the process of EMT in A549 by suppressing TGF-β1/Smad2/3 signaling.

The myofibroblast differentiation is characterized by altered expressions of α-SMA and proteins involved in ECM, cell migration, and proliferation (Rockey et al., 2015). After tissue injury, the repair process, including differentiation of fibroblasts into proliferating and contractile myofibroblasts, was initiated. When the normal repair process ends, myofibroblasts are regulated to undergo apoptosis; in pathological fibrosis, myofibroblasts accumulate abnormally in these tissues, increasing the synthesis of extracellular matrix, changing the tissue structure, and finally leading to fibrosis (Darby and Hewitson, 2007). In our study, BBJ could suppress fibroblast proliferation apparently. Fibroblast apoptosis was induced after 24 h intervention with 100 μl/ml BBJ. Furthermore, BBJ curbed differentiation and abnormal recruitment of fibroblasts caused by TGF-β1 by decreasing the upregulated p-Smad2/3 in NIH/3T3 and HPF. High expressions of α-SMA and collagen-I are the typical characteristics of myofibroblasts (Akamatsu et al., 2013). These were also found in RPLF cells, which were activated by BLM. No matter in HPF and NIH/3T3 incubated with TGF-β1 or activated RPLF, BBJ could suppress levels of α-SMA, vimentin, and collagen-I and increase levels of E-cadherin. Additionally, BBJ reversed the high expression of p-Smad2/3 caused by TGF-β1 in NIH/3T3 and HPF. Therefore, BBJ suppressed EMT and activated fibroblasts by blocking the TGF-β1/Smad2/3 signaling pathway.

Our hypothesis of an inhibitory effect of BBJ on pulmonary fibrosis was also supported by the *in vivo* results with BLM-induced lung fibrosis in mice. The BLM-induced lung fibrosis model is a widely used experimental model for lung fibrosis study. In the early phase (7–10 days), an acute inflammatory response is induced by BLM (Chaudhary et al., 2006). In the late phase (21–28 days), the inflammation decreases and fibrotic pathology changes occur and continue (Mouratis and Aidinis, 2011). Therefore, the mice were sacrificed on day 28 to investigate the preventive and inhibitory effects of BBJ on BLM-induced fibrosis, and the dose of BLM was from our preliminary experiments. In the research of anti-hepatic fibrosis activity, rats were gavage-fed daily with BBJ at a dose of 15 g/kg, and hence, we took 5 and 10 ml/kg as the dosages of BBJ to treat the lung fibrotic mice in the prevention model (Wang et al., 2013). And a higher dosage of BBJ was applied in the treatment model. Twenty-eight days after intratracheal injection of BLM, thickening of the lung interstitium, destruction of the alveolar structure, and collagen deposition happened in the lungs. However, continuous daily gavage of BBJ reversed these pathological changes in the dose of 10 ml/kg, whether in the prevention or treatment model. The involved mechanism was similar with altered protein expressions in cells, with remarkable decreased expressions of α-SMA, vimentin, collagen-I, and p-Smad3 and/or p-Smad2/3 as well as upregulated E-cadherin. Besides, collagen deposition was estimated by Masson staining and measuring of hydroxyproline content which decreased in the BBJ group.

Stat3 is a signaling molecule of signal transducers and activators of the transcription family. Our previous studies have elucidated that phytochemicals could suppress tumorigenesis and metastasis by blocking the Stat3 pathway (Li et al., 2019; Zhang et al., 2020). Recently, it is of great interest to manifest the role of Stat3 in fibrosis since abundant activation of Stat3 was confirmed in fibrotic pulmonary IPF patients (O'Donoghue et al., 2012). Also, Stat3 is identified as a positive regular of EMT, the mechanism of which activated Stat3 promotes Smad3 nucleus localization and further accelerates EMT (Junk et al., 2017). Hence, we detected Stat3 expression in the treatment mice model. BBJ and NTB downregulated p-Stat3 expression in fibrotic lung tissue induced by BLM. These results were consistent with previous research that Stat3 was involved in IPF. However, the underlying mechanism needs further study to be elucidated.

Collectively, our present work first revealed that BBJ could prevent and ameliorate IPF by modulating TGF-β1/Smad2/3 signaling. This important discovery not only provides support of dietary guidance for patients with pulmonary fibrosis but also proposes the possibility of juice rich in antioxidants as an accepted therapy for diseases. However, there are still some limitations in the study. One of the limitations in our research is that it is unknown how the ingredients of BBJ exerted antifibrosis activity collectively. Notably, the anti-lung fibrosis activity of BBJ based on evidence from epidemiological and clinical studies is an urgent need to address.

## 5 Conclusion

**FIGURE 7:**
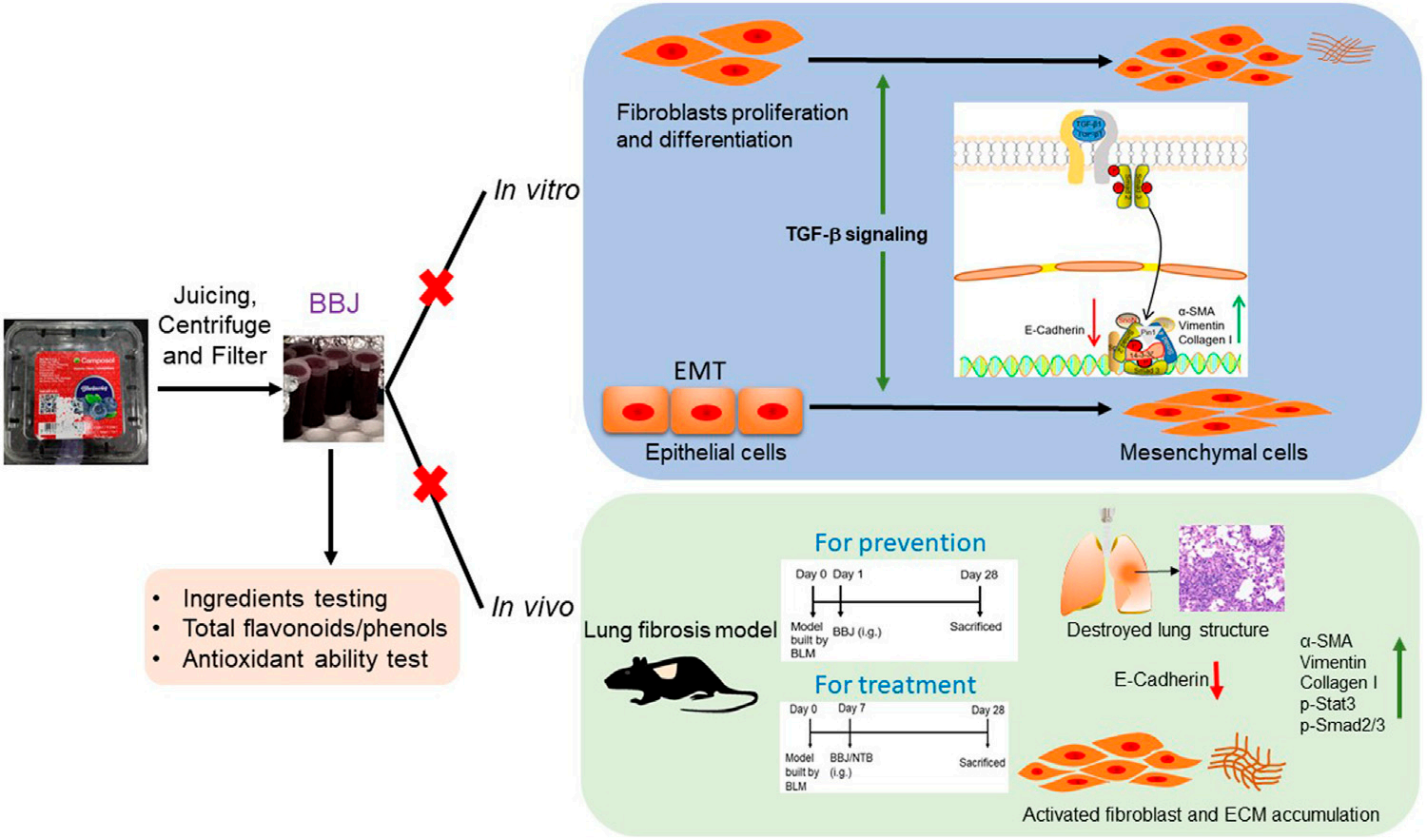
BBJ could inhibit lung fibrosis *in vivo* and *in vitro*. Followed by a series of processing, BBJ was utilized in assays *in vitro* and *in vivo*. BBJ was proven to inhibit EMT in A549. BBJ could also suppress differentiation of fibroblasts to myofibroblasts induced by TGF-β1 proved by the changes of related proteins. In addition, in lung fibrosis, the C57BL/6 mouse model built by BLM, BBJ could prevent and ameliorate lung fibrosis by suppressing the Smad2/3 pathway and Stat3.

Here, we firstly illustrated the effects of BBJ on pulmonary fibrosis *in vitro* and *in vivo*, as expressed in Figure 7. When incubated with TGF-β1-stimulated cells, including mouse embryo fibroblasts NIH/3T3, human pulmonary fibroblasts HPF, and alveolar epithelial A549, BBJ could accelerate the expression of epithelial mark E-cadherin and repress expressions of the mesenchymal markers α-SMA and vimentin, hindering the process of EMT. Likewise, the same changes were found in RPLF. Furthermore, BBJ exerted its anti-lung fibrosis effect by preventing cell migration and TGF-β1/Smad2/3 pathway activation. In BLM-induced pulmonary fibrosis mice, BBJ ameliorated the distortion of normal architecture by reducing collagen deposition and inhibiting the TGF-β1/Smad2/3 pathway. All these results indicated comprehensively that BBJ could attenuate lung fibrosis.

## Data Availability

The original contributions presented in the study are included in the article/Supplementary Material, further inquiries can be directed to the corresponding authors.
