# Supplementary material for: Blueberry Juice Attenuates Pulmonary Fibrosis via Blocking the TGF-β1/Smad Signaling Pathway
Source: Front Pharmacol. 2022 Mar 28;13:825915. doi: 10.3389/fphar.2022.825915 (PMC8996108; doi:10.3389/fphar.2022.825915)
Supplement: Supplementary file 1 [file DataSheet1.docx]

Supplementary Material

Blueberry juice attenuates pulmonary fibrosis via blocking TGF-β1/Smad signaling pathway

**Yali Li^1,2,3^, Liqun Wang^2^, Qianyu Zhang^2^, Li Tian^2^, Cailing Gan^1^, Hongyao Liu^1^, Wenya Yin*^2^, Tinghong Ye*^1^**

^1^Sichuan University-University of Oxford Huaxi Joint Centre for Gastrointestinal Cancer, Frontiers Science Center for Disease-Related Molecular Network, State Key Laboratory of Biotherapy, West China Hospital, Sichuan University, Chengdu, Sichuan, 610041, China.

^2^West China School of Public Health and West China Fourth Hospital, Sichuan University, Chengdu, China.

^3^Prenatal Diagnosis Center, The Third Affiliated Hospital of Zhengzhou University - Maternal and Child Health Hospital of Henan Province, Zhengzhou, China.

* Corresponding authors:

Tinghong Ye, Email: yeth1309@scu.edu.cn; Wenya Yin, Email: yinwenya@scu.edu.cn

**Supplementary Table 1 The possible components in BBJ**

| No. | Name | Formula | Molecular Weight | RT [min] | Area (Max.) | mz Vault Best Match | mzCloud Best Match |
| --- | --- | --- | --- | --- | --- | --- | --- |
| 1 | α, α-Trehalose | C_12_H_22_O_11_ | 342.11592 | 0.731 | 58948106.36 | 81.4 | 90.3 |
| 2 | Bis(methylbenzylidene)sorbitol | C_22_H_26_O_6_ | 386.17263 | 6.269 | 64199486.64 | 83.8 | 99.6 |
| 3 | Bis(4-ethylbenzylidene) sorbitol | C_24_H_30_O_6_ | 414.20393 | 6.669 | 167484803.7 | 83.1 | 99.5 |
| 4 | Palmitic Acid | C_16_H_32_O_2_ | 273.26668 | 5.876 | 1982383670 |  | 97.5 |
| 5 | Stearic acid | C_18_H_36_O_2_ | 301.29769 | 6.358 | 47932119.98 | 62.8 | 97.7 |
| 6 | N, N-Dimethylsphingosine | C_20_H_41_NO_2_ | 327.31352 | 6.494 | 10846767.86 | 65.6 | 99.4 |
| 7 | Arachidic Acid | C_20_H_40_O_2_ | 329.32909 | 6.824 | 57975722.47 |  | 94.6 |
| 8 | 2-Amino-1,3,4-octadecanetriol | C_18_H_39_NO_3_ | 317.29265 | 5.902 | 126528204.7 |  | 91.9 |
| 9 | DL-Arginine | C_6_H_14_N_4_O_2_ | 174.1116 | 0.668 | 136262705.4 | 92.9 | 99.7 |
| 10 | Leucine | C_6_H_13_NO_2_ | 131.09448 | 1.246 | 11833544.72 | 80 | 93.5 |
| 11 | 2-[2-(2,5-dichloroanilino)-2-oxoethyl]-N-(3,5-dimethylphenyl)-3-oxotetrahydro-1(2H)-pyrazinecarboxamide | C_21_H_22_Cl_2_N_4_O_3_ | 448.10008 | 4.522 | 43185329.84 | 90.6 | 73.6 |
| 12 | Trolox | C_14_H_18_O_4_ | 250.12039 | 6.691 | 2860626.301 |  | 92.6 |
| 13 | Choline | C_5_H_13_NO | 103.09961 | 0.699 | 193643284.8 | 76.1 | 99.8 |
| 14 | trans-3-Indoleacrylic acid | C_11_H_9_NO_2_ | 187.06322 | 2.885 | 13182374.03 | 91.7 | 93.6 |
| 15 | (±)-Abscisic acid | C_15_H_20_O_4_ | 264.136 | 5.133 | 10070381.51 | 91.2 | 97.8 |
| 16 | Butylparaben | C_11_H_14_O_3_ | 194.09421 | 6.091 | 4080414.68 |  | 94.2 |
| 17 | Ferulic acid | C_10_H_10_O_4_ | 194.05772 | 3.046 | 6431219.501 | 91.9 | 98.8 |
| 18 | Chlorogenic acid | C_16_H_18_O_9_ | 354.09477 | 3.262 | 17601567.88 | 90.4 | 99.7 |
| 19 | (1S,3R,4R,5R)-1,3,4-trihydroxy-5-{[(2E)-3-(4-hydroxy-3-methoxyphenyl) prop-2-enoyl]oxy}cyclohexane-1-carboxylic acid | C_17_H_20_O_9_ | 368.11052 | 3.978 | 4247321.204 | 87.3 | 98.9 |
| 20 | Rutin | C_27_H_30_O_16_ | 610.15333 | 4.174 | 32382263.32 | 95.2 | 96.6 |
| 21 | Quercetin | C_15_H_10_O_7_ | 302.04234 | 4.279 | 26547456.25 | 53.9 | 99.9 |
| 22 | Quercetin-3β-D-glucoside | C_21_H_20_O_12_ | 464.09531 | 4.28 | 256553976 | 96.5 | 99.3 |
| 23 | Gluconic acid | C_6_H_12_O_7_ | 196.0582 | 0.723 | 109424823.1 | 95.8 | 98.8 |
| 24 | 3-[2-(β-D-Glucopyranosyloxy)-4-methoxyphenyl] propanoic acid | C_16_H_22_O_9_ | 358.12611 | 3.325 | 2178489.476 | 79.9 | 97.9 |
| 25 | 3-[4-(beta-D-Glucopyranosyloxy)-6-methoxy-1-benzofuran-5-yl] propanoic acid | C_18_H_22_O_10_ | 420.10301 | 5.839 | 11588729.62 |  | 97.7 |
| 26 | 1-(β-D-Glucopyranosyloxy)-7-methyl-1,4a,5,6,7,7a-hexahydrocyclopenta[c]pyran-4-carboxylic acid | C_16_H_24_O_9_ | 360.14177 | 4.253 | 19590048.56 | 90.7 | 86.2 |
| 27 | (2R,3S,4S,5R,6R)-2-({[(2R,3R,4R,5S)-3,4-dihydroxy-5-(hydroxymethyl) oxolan-2-yl] oxy}methyl)-6-{[(2E)-3,7-dimethylocta-2,6-dien-1-yl]oxy}oxane-3,4,5-triol | C_21_H_36_O_10_ | 465.25707 | 5 | 14387341.8 | 81.5 | 96.7 |
| 28 | 5-({[3-chloro-5-(trifluoromethyl)-2-pyridyl] methyl} thio)-4-pentyl-4H-1,2,4-triazol-3-ol | C_14_H_16_ClF_3_N_4_OS | 380.07182 | 0.711 | 88822236.38 |  | 91.4 |
| 29 | Isophorone | C_9_H_14_O | 138.10431 | 5.663 | 7543752.078 | 80.4 | 97.2 |
| 30 | 2-(Methylsulfonyl)-3-(pyrazin-2-ylamino) acrylonitrile | C_8_H_8_N_4_O_2_S | 224.03557 | 6.118 | 3923257.408 |  | 97.3 |

**Supplementary Table 1 The possible components in BBJ**

One gram of BBJ was weighed precisely and extracted in 2 mL menthol for 30 min. After centrifuged, the superant was volumed to 10 mL, filtered by 0.22μm membrane filters, and then detected by LC/MS. We matched the results with the possible substances in BBJ from mzVauld and mzCloud databases in determination system.

**Supplementary Figure 1.** **The total ion chromatography result in BBJ components determination**

The possible substances in BBJ were analyzed by LC/MS. The detective condition was set as followed: ion source: H-ESI; source temperature: 320℃; detection mode: Full Scan-dd MS2; scanning range: 100-800 (m/z); Vaporizer Temp: 350℃; spray voltage: 3.5 kv positive ion, 3.2 kv negative ion; sheath gas: 50 Arb; Aux Gas: 15 Arb; intensity threshold: 1.0 x 10^4^.

**
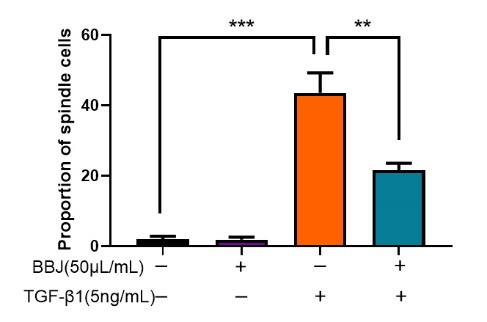
**

**Supplementary Figure 2. Proportion of spindle A549 cells**

Starved for 6h with serum-free medium, fresh medium with 5 ng/mL TGF-β1 were replaced. One hour later, 50 μL/mL BBJ was added. After for 24 h, cell morphology of A549 was observed by microscope (10x). Three visual fields for each group were chosen to count the proportion of spindle cells. The result was statistically analyzed. **P*<0.05; ***P*<0.01; ****P*<0.001 versus control group or TGF-β1 group.

**
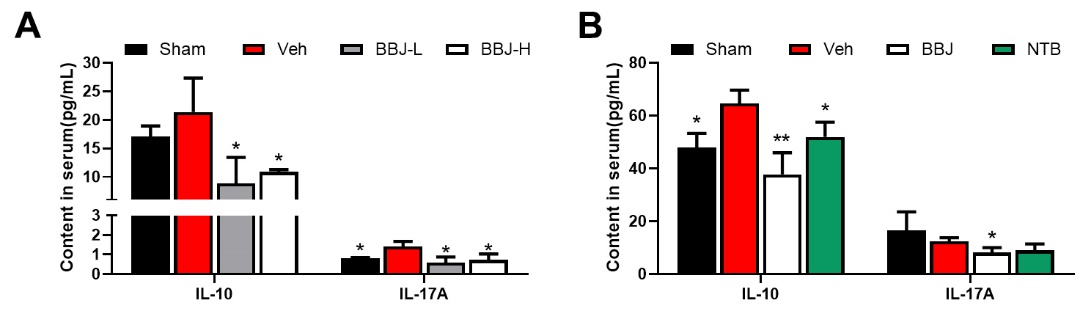
**

**Supplementary Figure 3.** **The levels of IL-10 and IL-17A in serum of BLM-induced lung fibrosis mouse.**

**(A)** For prevention model, fibrosis model was built by tracheal injection of BLM on Day 0. Next day, vehicle and BBJ-fed groups were administrated by saline and BBJ for 4 weeks, separately. The levels of IL-10 and IL-17A in serum were detected by Th1/Th2/Th17 kit. **(B)** In treatment model, fibrosis model was built by tracheal injection of BLM on Day 0. On Day 7, vehicle, BBJ-fed and NTB-fed groups were administrated by saline, BBJ and NTB for 3 weeks. The levels of IL-10 and IL-17A in serum were detected by Th1/Th2/Th17 kit through flowcytometry. **P*<0.05; ***P*<0.01; ****P*<0.001 compared with vehicle group.

**Original protein bands**

**Figure 1**

**β-actin**

**
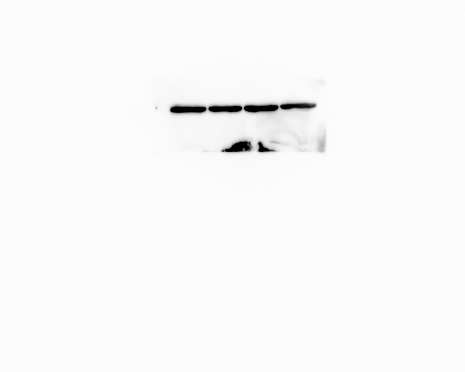
**

**cleaved Caspase3**

**
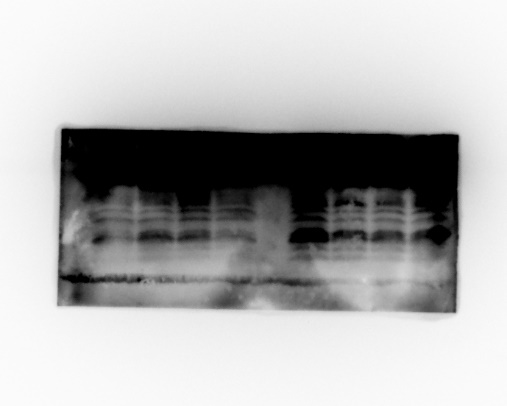
**

**Figure 2**

- **NIH/3T3**

**α-SMA**

**
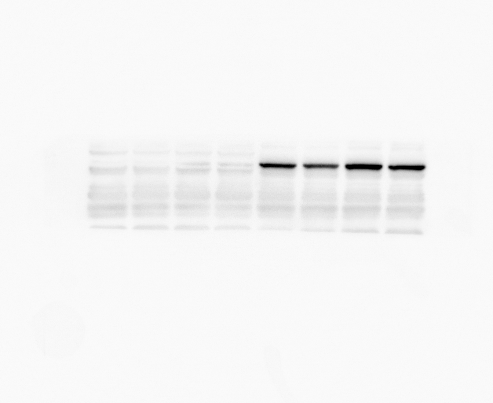
**

**Collagen I**

**
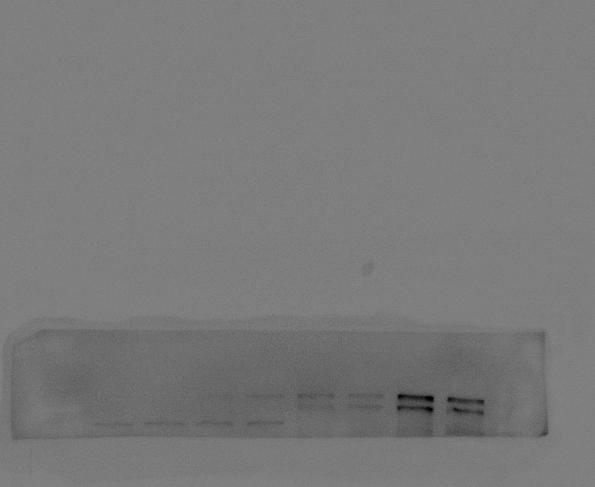
**

**Vimentin**

**
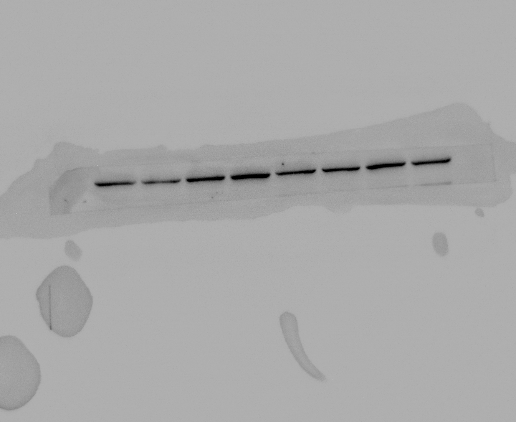
**

**p-Smad2/3**

**
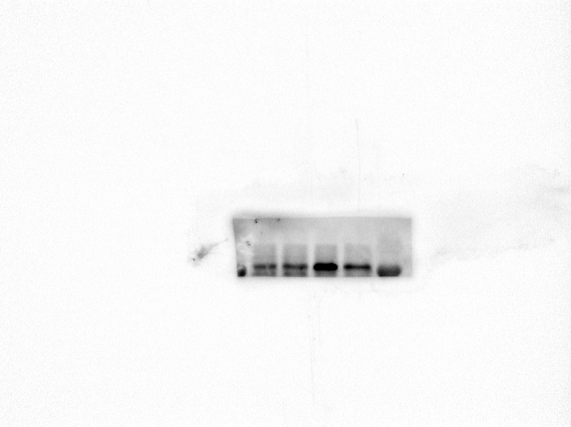
**

**Smad2/3**

**
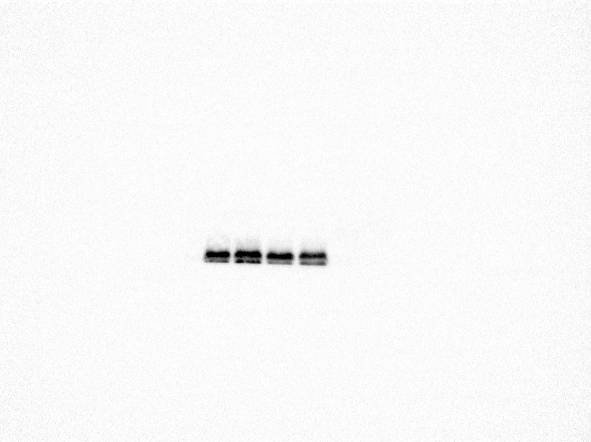
**

**β-actin**

**
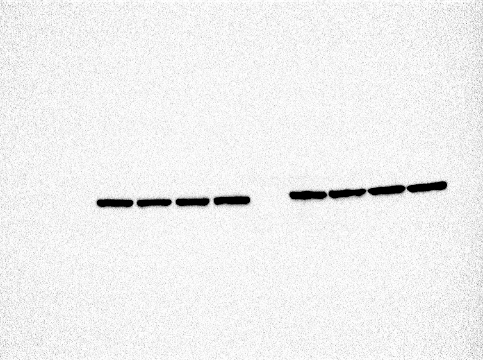
**

- **HPF**

**α-SMA**

**
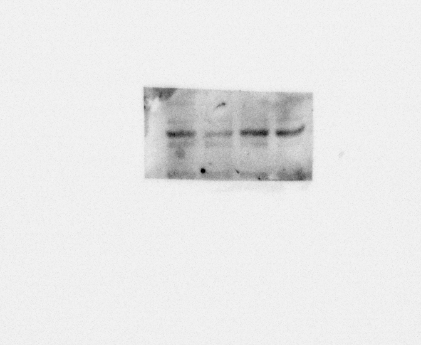
**

**E-Cadherin**

**
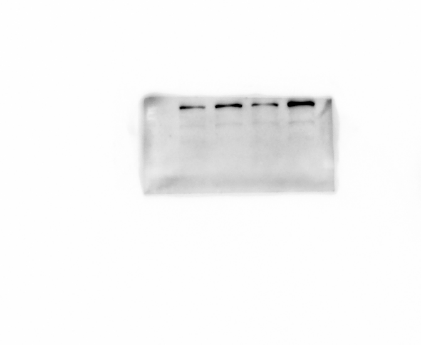
**

**Collagen I**

**
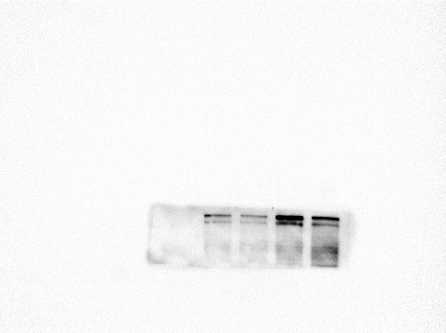
**

**Vimentin**

**
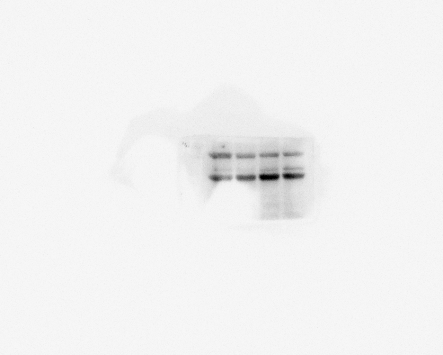
**

**p-Smad2/3**

**
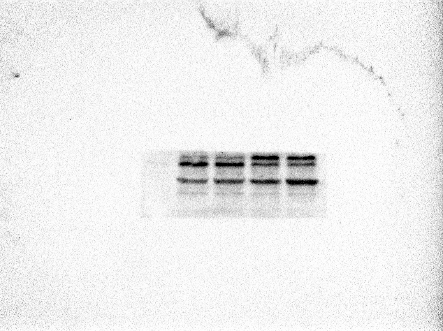
**

**Smad2/3**

**
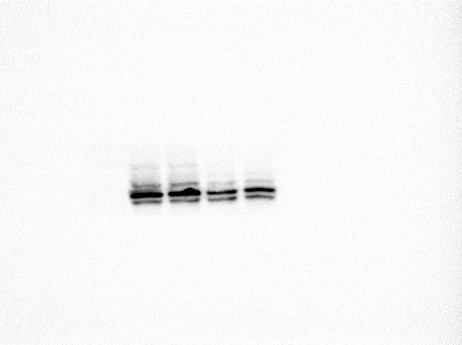
**

**β-actin**

**
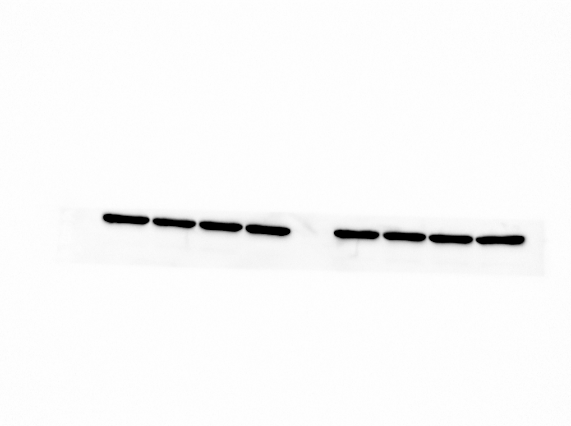
**

**Figure 3**

**α-SMA**

**
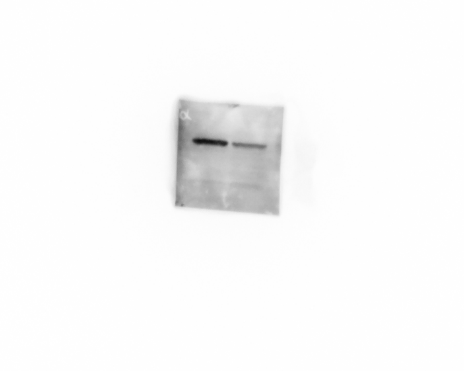
**

**E-Cadherin**

**
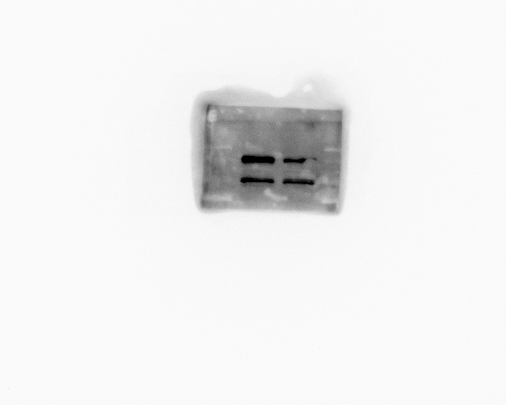
**

**Collagen I**

**
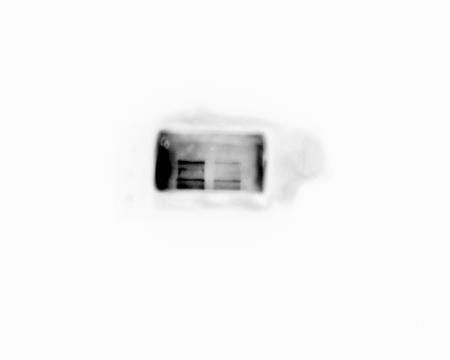
**

**Vimentin**

**
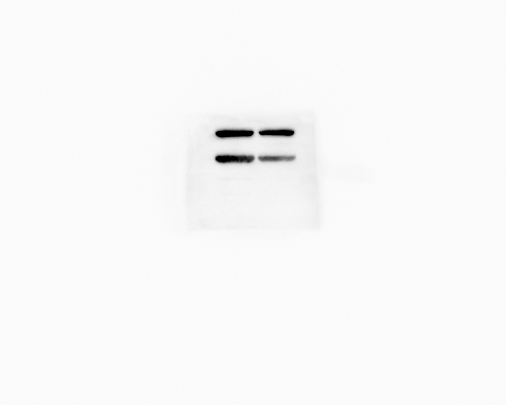
**

**β-actin**

**
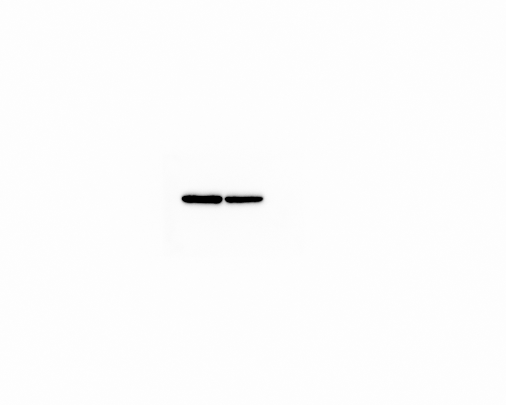
**

**Figure 4**

**α-SMA**

**
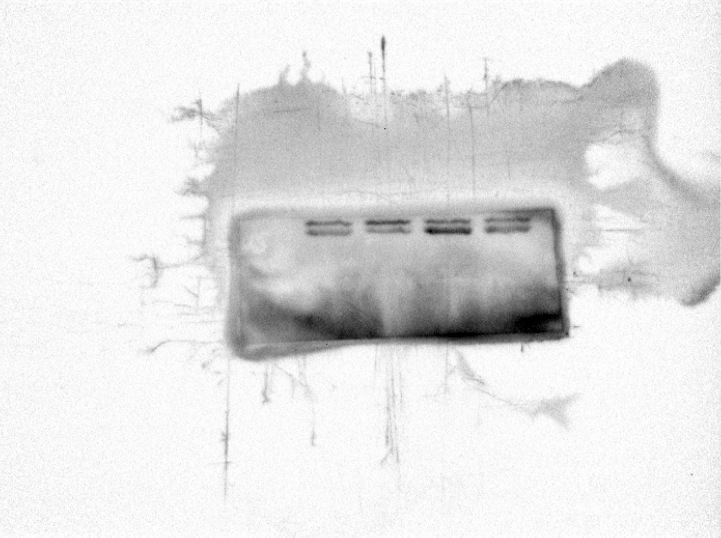
**

**E-Cadherin**

**
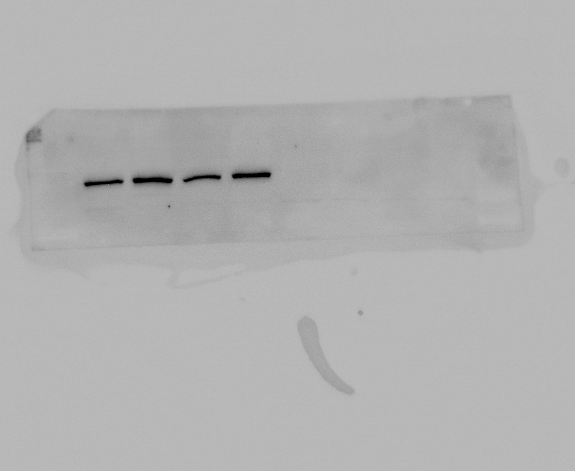
**

**Vimentin**

**
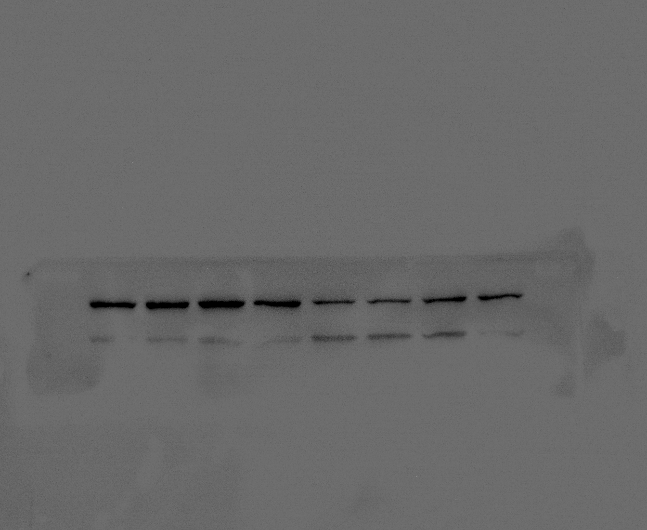
**

**p-Smad2/3**

**
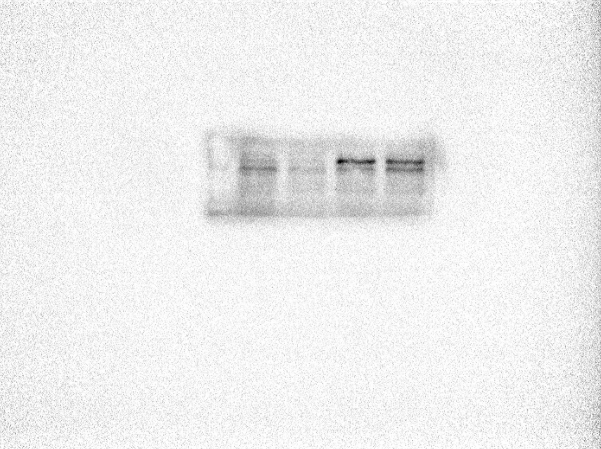
**

**Smad2/3**

**
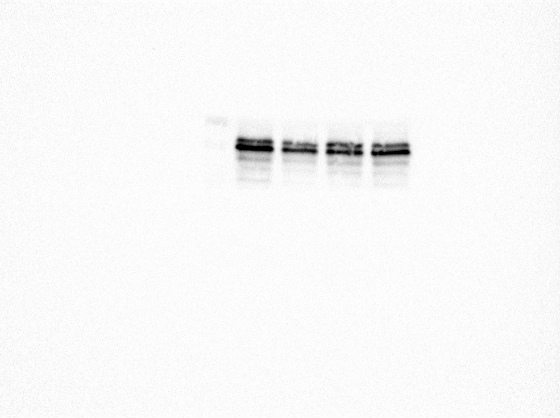
**

**β-actin**

**
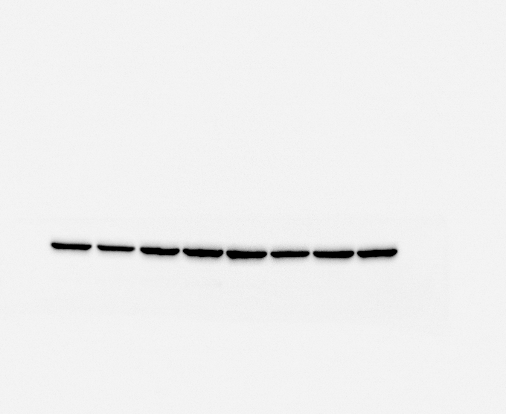
**

**Figure 5**

**
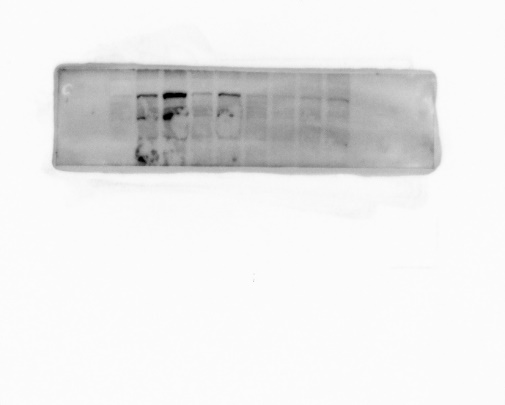
Collagen I**

**α-SMA**

**
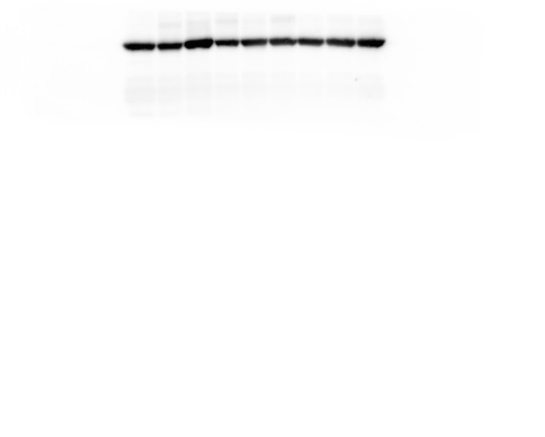
**

**Vimentin**

**
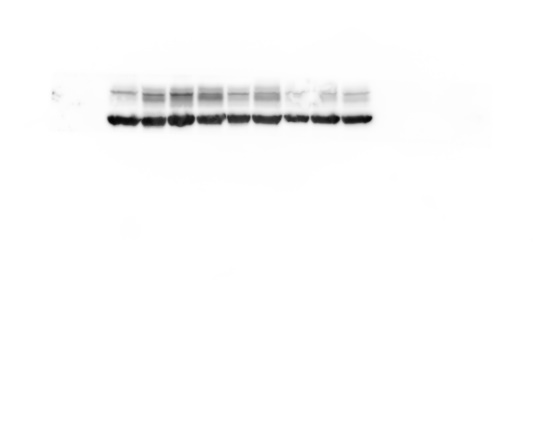
**

**β-actin**

**
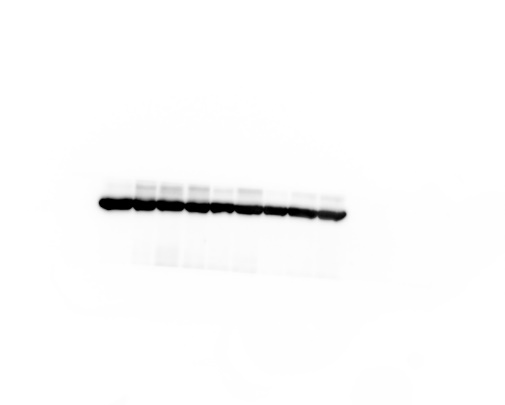
**

**Figure 6**

**α-SMA**

**
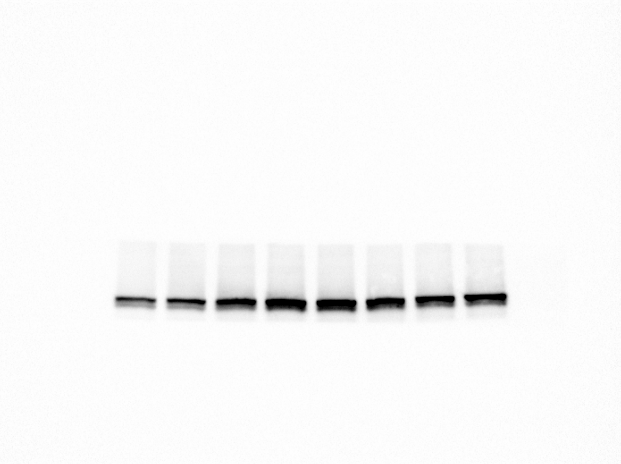
**

**E-Cadherin**

**
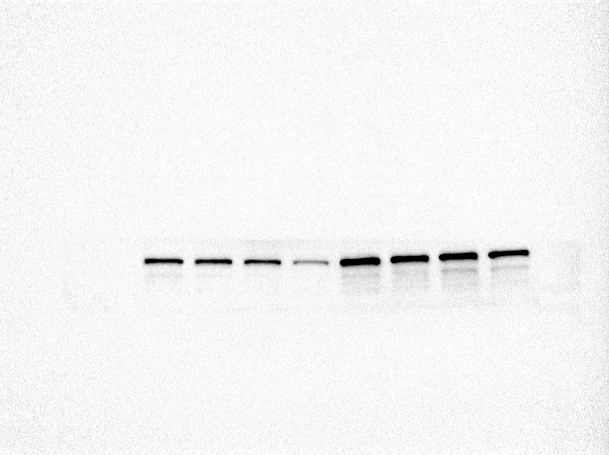
**

**Vimentin**

**
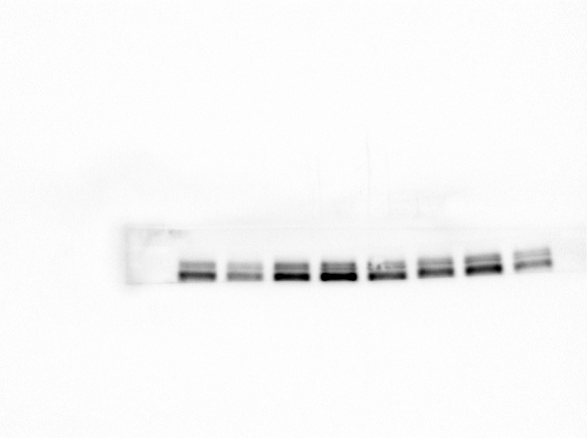
**

**Collagen I**

**
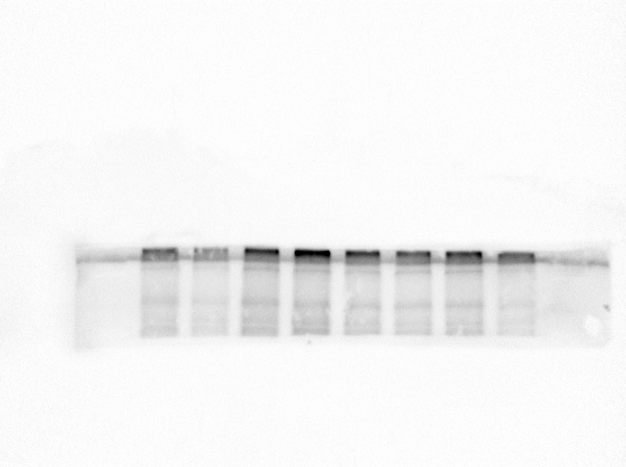
**

**p-Smad2/3**

**
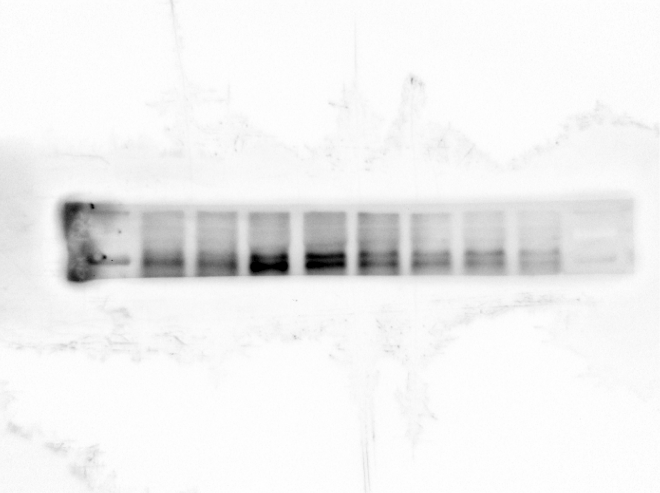
**

**Smad2/3**

**
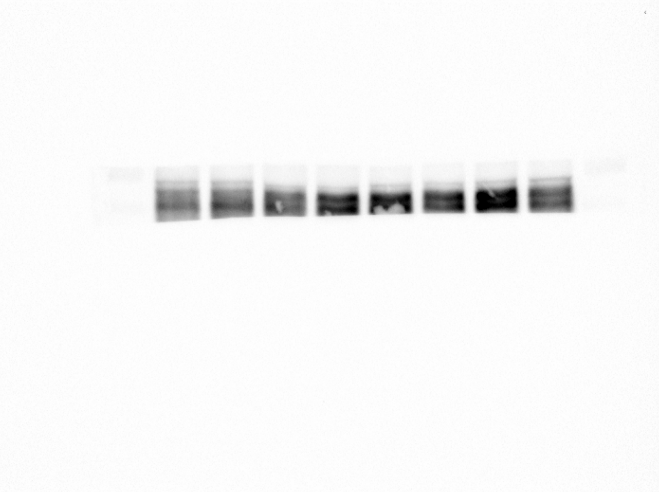
**

**p-Stat3**

**
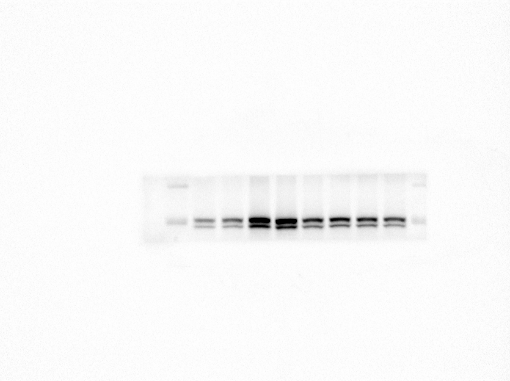
**

**Stat3**

**
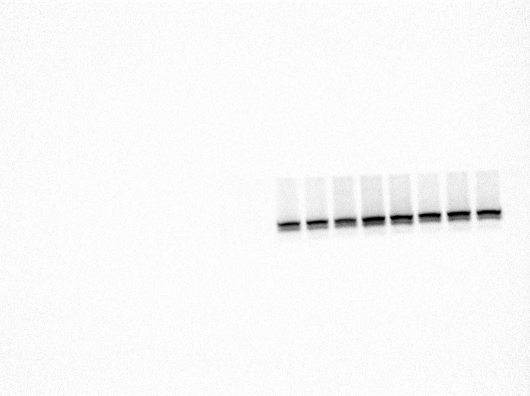
**

**β-actin**

**
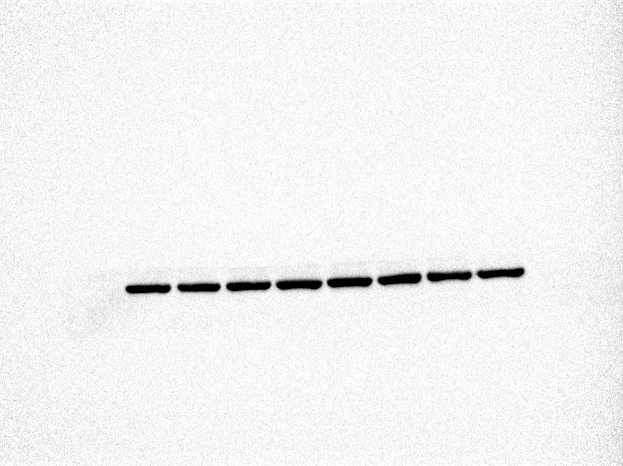
**
